# Pilot longitudinal integrated transcriptomic–metabolomic study reveals immune and metabolic signatures in non-hospitalized healthcare workers with long COVID

**DOI:** 10.3389/fcimb.2026.1808564

**Published:** 2026-06-04

**Authors:** Estefanía Espín, Chengliang Yang, Casey P. Shannon, Abhinav K. Checkervarty, Sehyeon Kim, Linda Lapp, Sara Assadian, Brian Grunau, David M. Goldfarb, Jacob Hutton, Tao Huan, Scott J. Tebbutt

**Affiliations:** 1 Prevention of Organ Failure (PROOF) Centre of Excellence, Providence Research. St. Paul’s Hospital, Vancouver, BC, Canada; 2 Centre for Heart Lung Innovation, The University of British Columbia, Providence Research, Vancouver, BC, Canada; 3 Department of Chemistry, Faculty of Science, The University of British Columbia, Vancouver, BC, Canada; 4 Department of Anesthesiology, Pharmacology and Therapeutics, Faculty of Medicine, The University of British Columbia, Vancouver, BC, Canada; 5 Department of Emergency Medicine, Faculty of Medicine, The University of British Columbia, St. Paul’s Hospital, Vancouver, BC, Canada; 6 British Columbia Emergency Health Services, Provincial Health Services Authority, Vancouver, BC, Canada; 7 Department of Pathology and Laboratory Medicine, The University of British Columbia, Vancouver, BC, Canada

**Keywords:** biomarkers, long covid, metabolomics, outpatients, transcriptomics

## Abstract

**Introduction:**

Long COVID affects hundreds of millions of individuals worldwide, yet its underlying biological mechanisms remain incompletely understood, and the absence of validated biomarkers continues to limit diagnosis and clinical management. Most biomarker studies have focused on hospitalized patients with severe disease, leaving non-hospitalized populations, particularly healthcare workers, who are at high occupational risk, underrepresented. This gap may constrain the identification of biomarkers relevant to milder but persistent post-acute phenotypes.

**Methods:**

We performed integrated transcriptomic and metabolomic profiling in a longitudinal cohort of non-hospitalized healthcare workers with long COVID (N = 12), primarily presenting with fatigue and brain fog, and matched controls who recovered from SARS-CoV-2 infection without sequelae (N = 35). Whole-blood RNA extracted from PAXgene tubes was profiled using the NanoString nCounter PanCancer Immune Panel. Serum metabolites collected pre- and post-infection were analyzed using untargeted ultra-high-performance liquid chromatography–mass spectrometry. Differential expression and metabolite abundance were assessed using linear models with false discovery rate correction. Significant features were integrated using network- and pathway-based approaches to identify coordinated immune–metabolic alterations in long COVID.

**Results:**

Transcriptomic analysis identified 63 differentially expressed genes, including neutrophil-associated markers such as *S100A8* and *LY96*, consistent with activation of innate inflammatory pathways. Metabolomic profiling identified 24 annotated metabolites, with oxoglutarate exhibiting a distinct longitudinal trajectory, increasing in long COVID cases while decreasing in controls. Integrated network analysis highlighted central nodes (APP, RELA, ATF2, HLA-B) and revealed pathway-level convergence on necroptosis and serotonergic synapse signaling, suggesting coordinated immune–metabolic dysregulation rather than isolated gene-level effects.

**Discussion:**

These findings generate hypotheses regarding potential links between persistent innate immune activation, metabolic reprogramming, and neurocognitive or systemic symptoms in long COVID. The observed signatures suggest immune–metabolic perturbations involving neutrophil-associated inflammatory pathways and broader cellular stress responses. However, given cohort size, platform-specific constraints, and cross-cohort heterogeneity, these signals should be interpreted at the pathway level and considered candidate mechanisms requiring validation in larger, independent cohorts of non-hospitalized individuals.

## 1 Introduction

Long COVID (LC) is defined by the World Health Organization (WHO) as a condition in which symptoms persist or newly develop three months after acute SARS-CoV-2 infection, last for at least two months, and cannot be explained by alternative diagnoses (World Health Organization, 2022). In 2024, the National Academies of Sciences, Engineering, and Medicine (NASEM) further characterized LC as an infection-associated chronic condition lasting at least three months, with continuous, relapsing–remitting, or progressive courses that can affect one or more organ systems (Ely et al., 2024).

The pooled estimated global prevalence of long COVID (LC) among individuals with prior SARS-CoV-2 infection is 36%, according to a recent meta-analysis encompassing 144 studies (Hou et al., 2025). Despite its substantial burden and multisystemic presentation, affecting the nervous, cardiovascular, immune, endocrine, reproductive, and gastrointestinal systems, LC remains difficult to diagnose and is frequently underrecognized or misdiagnosed (Greenhalgh et al., 2022; Al-Aly et al., 2024). No specific biomarkers currently exist for LC, and standard clinical tests often yield normal results, particularly among non-hospitalized individuals, who remain underrepresented in research. Although LC risk is well established in hospitalized patients, most cases arise following mild acute infection (Choutka et al., [[NoYear]]; Davis et al., 2023). This contributes to ongoing diagnostic uncertainty and significant gaps in care (Bell et al., 2021; Subramanian et al., 2022; Ose et al., 2024). Recent studies suggest that LC pathophysiology involves persistent viral reservoirs and immune dysregulation, leading to chronic inflammation, endothelial injury, and microvascular dysfunction (Yang et al., 2023). Transcriptomic analyses have identified inflammatory gene signatures in blood, consistent with tissue and plasma evidence of intermittent SARS-CoV-2 viral antigen or RNA detection up to a year post-infection (Peluso and Deeks, 2024). At the metabolic level, mitochondrial dysfunction and altered energy metabolism have been linked to fatigue and post-exertional malaise, core symptoms shared with myalgic encephalomyelitis/chronic fatigue syndrome (ME/CFS) (Peluso and Deeks, 2024). However, the temporal dynamics and diagnostic relevance of these immune–metabolic alterations remain unclear, particularly in non-hospitalized populations.

Healthcare workers represent another understudied population. They experienced a higher risk of SARS-CoV-2 infection than the general population, and among those infected, the pooled prevalence of LC is estimated at 40%, with a mean follow-up of 22 weeks, and symptoms most commonly present as fatigue and neurological symptoms (Al-Oraibi et al., 2025). Recent large-scale community-based studies have demonstrated that commonly reported cognitive symptoms after COVID-19, including poor memory and difficulty concentrating (“brain fog”), are associated with objectively measurable deficits in memory, reasoning, and executive function (Hampshire et al., 2024).

To address these gaps, we performed an exploratory integration of transcriptomic and metabolomic profiling in non-hospitalized healthcare workers with LC, aiming to identify gene and metabolite signatures that could represent candidate biomarkers. We additionally conducted immune transcriptional profiling and cross-cohort validation using independent publicly available transcriptomic and metabolomic datasets to assess the reproducibility and biological relevance of the identified signatures. We also analyzed metabolomic profiles at baseline (pre-infection) and post-infection timepoints, providing preliminary longitudinal insights and partially addressing a limitation of previous studies that lacked pre-infection samples.

## 2 Materials and methods

### 2.1 Study population

We compared transcriptomic and metabolomic profiles between individuals with and without LC from the British Columbia (BC) cohort of the COVID-19 Occupational Risks, Seroprevalence, and Immunity among Paramedics in Canada (CORSIP) study, a prospective longitudinal cohort of adult paramedics (Grunau B, 2020). Between January 2021 and January 2023, paramedics aged ≥19 years were recruited in BC through staggered enrollment. Participants completed web-based sociodemographic and health questionnaires at enrollment and at 6-, 12-, 18-, and 24-month follow-ups. The present study was not part of the original CORSIP study design and emerged as an extension analysis near completion of cohort follow-up. Consequently, analyses were designed to maximize the use of available biospecimens and longitudinal data.

Seventeen LC cases were identified through the 24-month questionnaire. Cases were defined according to the WHO clinical case definition as participants reporting i) new symptoms lasting ≥2 months beginning after 2020, and ii) symptoms persisting from, or emerging within, 3 months of confirmed SARS-CoV-2 infection. Symptoms were assessed using standardized self-reported questionnaires administered at each follow-up visit. Controls consisted of participants with confirmed SARS-CoV-2 infection who did not report LC symptoms during the study period. SARS-CoV-2 infection was determined by a positive PCR or antigen test, detection of anti-SARS-CoV-2 nucleocapsid antibodies, or symptom-based diagnosis. Participants were excluded if they i) resided outside BC, where biospecimens were collected, ii) lacked confirmed SARS-CoV-2 infection, or iii) were missing required biospecimens.

Serum samples were collected at each study visit, processed, and stored at −70 °C to −80 °C until testing. Most participants were enrolled prior to SARS-CoV-2 infection and subsequently contracted the virus during longitudinal follow-up, as confirmed through questionnaires and health records. Consequently, many participants had paired pre- and post-infection serum samples available for longitudinal metabolomic analyses. Eligibility for metabolomic analyses required paired pre- and post-infection serum samples. Six LC cases met these criteria and were matched 1:1 to six controls using propensity scores generated with the MatchIt package (R v4.5.0). Nearest-neighbor matching was performed based on age, sex, and sampling time window relative to infection to maximize comparability between groups and account for temporal variation in metabolomic profiles.

Whole blood samples collected in PAXgene^®^ tubes (PreAnalytiX, Hombrechtikon, Switzerland) for transcriptomic profiling were obtained only at the final study visit (January 2023), resulting in a smaller subset of participants with available RNA samples. PAXgene samples were stored at 2–8 °C for up to five days before long-term storage at −70 °C to −80 °C, in accordance with manufacturer recommendations. Transcriptomic analyses included all eligible samples that passed RNA extraction and integrity quality control, resulting in 8 LC cases and 29 controls. Because the number of available LC RNA samples was limited, strict one-to-one matching was not feasible. Instead, controls were selected to ensure comparable age and sex distributions between groups. Due to differences in serum and whole blood sample availability and collection timepoints, not all LC cases had matching samples for both metabolomic and transcriptomic analyses. Demographic characteristics of metabolomic and transcriptomic case-control groups were compared using the Mann–Whitney U test for continuous variables and Fisher’s exact test for categorical variables to assess potential group differences (**Table 1**).

**Table 1:** Participant demographics and clinical characteristics for transcriptomic and metabolomic analyses.

Variable	All participants No. (%) (N = 47)^*^			Subset of participants in transcriptomics No. (%) (N = 37)			Subset of participants in metabolomics No. (%) (N = 12)		
	LC Case (N = 12)	Control (N = 35)	P value	LC Case (N = 8)	Control (N = 29)	P value	LC Case (N = 6)	Control (N = 6)	P value
Age, median (IQR), years	43.5 (34.2 to 51.2)	37.0 (32.5 to 45.5)	0.29	43.5 (39.5 to 48.8)	36.0 (32.0 to 44.0)	0.10	50.0 (36.0 to 52.8)	51.5 (42.2 to 57.8)	0.58
BMI, median (IQR), kg/m^2^	32.8 (28.3 to 35.5)	29.4 (27.2 to 35.6)	0.574	31.1 (28.3 to 40.4)	29.4 (26.4 to 35.2)	0.483	32.8 (29.8 to 34.0)	34.6 (29.2 to 36.2)	0.470
Sex									
Male	4 (33%)	21 (60%)	0.18	3 (38%)	18 (62%)	0.25	2 (33%)	3 (50%)	> 0.99
Female	8 (67%)	14 (40%)		5 (62%)	11 (38%)		4 (66%)	3 (50%)	
Race and ethnicity									
White	12 (100%)	29 (83%)	> 0.99	8 (100%)	23 (79%)	> 0.99	6 (100)	6 (100%)	> 0.99
White-Japanese	0	1 (3%)		0	1 (3%)		0	0	
White-Arab	0	1 (3%)		0	1 (3%)		0	0	
White-Southeast Asian	0	1 (3%)		0	1 (3%)		0	0	
Chinese	0	1 (3%)		0	1 (3%)		0	0	
Latin American	0	1 (3%)		0	1 (3%)		0	0	
Arab-Prefer to self-describe	0	1 (3%)		0	1 (3%)		0	0	
Comorbidities									
Hypertension	2 (17%)	4 (11%)	0.64	1 (13%)	3 (10%)	> 0.99	1 (17%)	1 (17%)	> 0.99
Diabetes	1 (8%)	1 (3%)	0.45	1 (13%)	0	0.20	0	1 (17%)	> 0.99
Asthma	3 (25%)	6 (17%)	0.67	2 (25%)	3 (10%)	0.25	2 (33%)	3 (50%)	> 0.99
Lung disease	0	1 (3%)	> 0.99	0	1 (3%)	> 0.99	0	0	> 0.99
Immune disease	0	0	> 0.99	0	0	> 0.99	0	0	> 0.99
Neurological condition	0	1 (3%)	> 0.99	0	1 (3%)	> 0.99	0	0	> 0.99
1st dose SARS-CoV-2 vaccine before infection	9 (75%)	26 (97%)	0.06	5 (63%)	20 (95%)	0.05	6 (100%)	6 (100%)	> 0.99
Infection_variant_timing^†^									
Omicron	9 (75%)	26 (97%)	0.08	5 (62%)	20 (95%)	0.052	6 (100%)	6 (100%)	> 0.99
Pre-Omicron	3 (25%)	1 (4%)		3 (38%)	1 (5%)		0	0	
Hospitalized due to SARS-CoV-2 infection	0	0	0.25	0	0	0.22	0	0	> 0.99
Prefer not to answer	1 (8%)	0		1 (13%)	0		0	0	
LC symptoms									
Fatigue	8 (67%)	0	NA	5 (63%)	0	NA	4 (67%)	0	NA
Brain fog	7 (58%)	0		4 (50%)	0		4 (67%)	0	
Cough	5 (42%)	0		3 (38%)	0		2 (33%)	0	
Memory impairments	4 (33%)	0		2 (25%)	0		2 (33%)	0	
Decreased energy to exercise	3 (25%)	0		1 (13%)	0		2 (33%)	0	
Other	3 (25%)	0		3 (38%)	0		1 (17%)	0	
Shortness of breath	3 (25%)	0		3 (38%)	0		0	0	
Post-exertion fatigue	2 (17%)	0		1 (13%)	0		1 (17%)	0	
Sore	2 (17%)	0		1 (13%)	0		1 (17%)	0	
Symptoms duration, months, median (IQR)	10.5 (7 to 13)	0		10.5 (3.75 to 17.5)	0		10.5 (9.25 to 11.75)	0	
Sample collected after infection, months, median (IQR)	NA	NA	NA	8.9 (5.5 to 25.4)	8.1 (5.3 to 9.3)	0.38	4.7 (3.4 to 5.7)	5.5 (4.2 to 6.2)	0.63
Sample collected before infection, months, median (IQR)	NA	NA	NA	NA	NA	NA	-7.2 (-10.0 to -3.1)	-5.9 (-6.2 to -5.3)	> 0.99

*The total sample size (N = 47) includes 29 controls and 8 LC cases for transcriptomics, and 6 controls and 6 LC cases for metabolomics. Two LC participants were included in both assays, having both PAXgene (transcriptomics) and serum (metabolomics) samples.

^†^ Infections were considered Pre-Omicron if they occurred before December 1, 2021, based on the first reported Omicron case in Ontario in late November 2021.

^†^ Continuous variables are presented as medians (interquartile range) and compared using the Mann–Whitney U test; categorical variables are presented as counts (percentages) and compared using Fisher’s exact test.

Ethics approval was obtained from the University of British Columbia (H20-03620) and the University of Toronto (#40435). Electronic informed consent was obtained from all participants prior to enrollment. All procedures were conducted in accordance with institutional guidelines and regulations, including the Declaration of Helsinki and the Tri-Council Policy Statement: Ethical Conduct for Research Involving Humans (TCPS 2).

### 2.2 External datasets for cross-cohort validation

To evaluate the reproducibility of our findings in the context of the small sample size and exploratory nature of this pilot study, we assessed differential expression, directionality consistency, and signature correlations using independent publicly available LC datasets defined according to WHO criteria. Due to the limited availability of external cohorts closely matching our study design, it was not possible to identify validation datasets with the same combination of whole-blood samples, non-hospitalized LC participants, and NanoString PanCancer Immune Profiling data. Although single-cell RNA sequencing datasets were publicly available, these were considered methodologically distinct from the targeted immune-panel approach used in the present study. Therefore, we selected the publicly available datasets most comparable to our cohort characteristics and analytical platform.

Transcriptomics Cohort A (GSE224615) included 27 LC cases and 16 recovered controls. Acute-phase hospitalization occurred in 26% of LC participants and 12.5% of controls. Bulk RNA sequencing of peripheral blood mononuclear cells (PBMCs) was performed approximately 8 months post-infection (Yin et al., 2024). Transcriptomics Cohort B (GSE275334) included 15 individuals with LC and 18 healthy controls; hospitalization status was not reported. PBMC samples collected at least 3 months post-infection were profiled using the NanoString Immune Exhaustion panel (Eaton-Fitch et al., [[NoYear]]).

The metabolomics cohort included 15 LC cases and 33 recovered controls, all previously hospitalized. Plasma samples collected approximately 2 years post-infection were analyzed using direct-injection mass spectrometry and reverse-phase LC–MS/MS targeted profiling (López-Hernández et al., 2023).

### 2.3 RNA extraction

Whole-blood samples were collected in PAXgene Blood RNA Tubes (PreAnalytiX, Qiagen/BD) and stored at -80 °C until processing. Prior to RNA extraction, tubes were thawed at room temperature for 2 hours. All procedures were conducted under RNase-free conditions to minimize RNA degradation. Because human blood may contain infectious agents, all sample handling was performed under Biosafety Level 2 (BSL-2) conditions in a Class II Biosafety Cabinet with appropriate protective equipment (gloves, lab coat, and eye protection). Reagent bottles for buffers BM1–BM4 were inspected for precipitates; ethanol was added to BM3 and BM4, and DNase I stock solution was prepared in 550 µL RNase-free water and stored at 4 °C for up to 6 weeks. Five milliliters of blood from each PAXgene tube were transferred to a 15 mL conical tube in the biosafety cabinet. Conical tubes were centrifuged at 4,600 × g for 10 minutes at 4 °C. The supernatant was carefully removed, and the pellet was resuspended in 4 mL RNase-free water and vortexed until fully dissolved. After a second centrifugation under identical conditions, the pellet was resuspended in 350 µL BM1 buffer and transferred to 2 mL processing tubes for automated purification on the QIAcube.

Reagents for the QIAcube were prepared for 12 samples as follows: 558 µL proteinase K in tube A, 806 µL buffer RDD with 115 µL DNase I in tube B, and 1,177 µL BR5 elution buffer divided into two 588.5 µL aliquots in tube C. Twelve 1.5 mL microcentrifuge tubes were labeled, and rotor adapters for the QIAcube were assembled with shredder and spin columns in the designated positions. RNA extraction was performed in two automated steps on the QIAcube. Part A included automated processing of the lysed blood samples with proteinase K, DNase I treatment, and column-based purification. After Part A, reagent bottles were closed, empty processing tubes and spin columns were discarded, and samples were transferred to a shaker to ensure proper mixing. Part B involved RNA elution (70 µL) into 1.5 mL tubes, with a 3 µL aliquot separated into an Agilent strip tube; all samples were stored at -80 °C.

RNA concentration and purity were measured immediately after extraction using a NanoDrop ND-1000 spectrophotometer (Thermo Fisher Scientific, Wilmington, DE) with 1.5 µL of sample, blanked with BR5 elution buffer, and values were recorded. RNA integrity was assessed using the Agilent 2100 Bioanalyzer with the RNA 6000 Nano Kit (Agilent Technologies, Santa Clara, CA). RNA aliquots were retrieved from -80 °C, thawed on ice, and gently mixed by pipetting to avoid shearing. For chip preparation, 12 µL of gel-dye mix was loaded into designated wells of the RNA Nano chip, which was then primed using the provided priming station to ensure uniform gel distribution. Samples were prepared by mixing 1 µL RNA with 9 µL RNA loading buffer, denatured at 70 °C for 2 minutes, and immediately placed on ice. Five microliters of each sample were loaded into the primed chip wells alongside RNA ladder and marker wells. The chip was inserted into the Agilent 2100 Bioanalyzer, and the “RNA Nano” assay protocol was launched using the 2100 Expert Software. RNA fragments were separated by size via microcapillary electrophoresis and detected by fluorescence. Electropherograms were examined to assess RNA quality, and the RNA Integrity Number (RIN), ranging from 1 (degraded) to 10 (intact), was used to evaluate integrity. Only samples with RIN ≥ 7 were considered suitable for downstream transcriptomic analyses.

### 2.4 Transcriptomic profiling

Gene expression profiling was performed using the NanoString nCounter PanCancer Immune Profiling Panel (NanoString Technologies, Seattle, WA), which measures 770 immune-related transcripts, following the manufacturer’s instructions (MAN-10056-05). This targeted gene expression platform was selected due to its reproducible quantification of immune-related genes, lower technical variability, and reduced computational burden compared with whole-transcriptome profiling, which is advantageous given the small sample size and hypothesis-generating objective. The focus on an immune-oriented panel was supported by our prior scoping review identifying immune-related molecules as predominant candidate biomarkers in LC (Espín et al., 2023). In addition, 20 candidate genes identified from the biomarker scoping review were incorporated as a CodeSet Plus add-in: *CMV UL83, EBV LMP2, SARS-CoV-2_N, SARS-CoV-2_M, SARS-CoV-2_S, CST3, FKBP14, SRC, STAM, USP8, KDR, ACE2, ADIPOQ, APOA1, CXCL8, HLA-DRB1, IL33, MCU, NKG7*, and *VWF*. All RNA handling was performed under RNase-free conditions using dedicated RNase-free consumables, and work surfaces were disinfected to minimize RNase contamination.

For hybridization, 100 ng of total RNA per sample was used. Reporter CodeSet, Reporter Plus, and hybridization buffer were combined directly in the Reporter CodeSet tube to prepare a master mix (final per-reaction volumes: 3 µL Reporter CodeSet, 2 µL Reporter Plus, 5 µL hybridization buffer). In parallel, the Capture ProbeSet and Capture Plus reagents were mixed by gentle inversion, and 3 µL of the combined capture solution was added to each reaction. Five microliters of RNA sample were then added to each well, bringing the final hybridization reaction volume to 18 µL. Reagents were handled by gentle inversion or flicking to avoid probe shearing, and samples were protected from light during preparation.

Reactions were assembled in hybridization strip tubes, sealed, and briefly centrifuged at ≤1,000 rpm to collect contents at the bottom of the tubes. Samples were incubated in a thermal cycler with a heated lid set to 70 °C and a reaction temperature of 65 °C for 16–24 hours. After incubation, tubes were cooled to 4 °C and processed within 24 hours to minimize background signal.

On the following day, hybridized samples were transferred to the nCounter Prep Station and processed with the nCounter Master Kit using the High Sensitivity protocol. A single-use sample cartridge was equilibrated to room temperature, loaded with processed samples, and sealed immediately after Prep Station completion. Cartridges were scanned on the nCounter Digital Analyzer, with data collected at the maximum field of view.

For data acquisition, a merged Reporter Library File was used to ensure detection of both the PanCancer Immune Profiling Panel probes and the 20-gene Plus add-in targets. This file was generated by combining the RLF from the core CodeSet with the add-in library file provided for the Plus panel, following the manufacturer’s instructions. All steps were performed under RNase-free conditions, and samples were protected from light to prevent photobleaching. All procedures involving human RNA were performed under Biosafety Level 2 (BSL-2) conditions.

#### 2.4.1 Transcriptomic data preprocessing

Raw gene expression data were obtained using the NanoString nCounter platform. Data quality was evaluated in nSolver Analysis Software (NanoString Technologies) using standard quality control (QC) metrics, including imaging efficiency, binding density, positive control linearity, and limit of detection. All samples passed QC thresholds.

Background correction was performed in nSolver using negative control probes. A threshold was calculated as the mean of the negative controls plus two standard deviations, and counts below this threshold were adjusted. Gene expression data were normalized sequentially using positive control normalization and CodeSet content (housekeeping genes) normalization in nSolver. Positive control normalization factors were calculated using the geometric mean of six synthetic DNA control probes and applied to each lane. Housekeeping gene normalization factors were computed from the geometric mean of stable housekeeping genes, excluding low-count genes (<100 counts; *CC2D1B, ZKSCAN5, GPATCH3, DNAJC14*), and applied to all probes. All samples passed normalization QC and were retained for downstream analyses. Normalized counts were exported from nSolver for analysis in R.

#### 2.4.2 Immune transcriptional profiling

Given that *S100A8* (monocyte-associated) and *KIR2DL1* (NK cell–related inhibitory receptor) were both significantly differentially expressed and exhibited a fold change of |FC| > 1.5, they were selected for immune transcriptional profiling using predefined NanoString immune functional modules (NK cell function, cytotoxicity, neutrophil inflammation, and KIR signaling pathways, **Supplementary Table 1**) to enhance biological interpretability.

Immune cell-type and functional activity scores (NK cell abundance and activity, cytotoxic T-cell activity, monocyte/macrophage activity, and neutrophil-associated transcriptional activity) were derived as gene signature scores. For each sample, scores were calculated as the mean of normalized expression values across genes within each predefined NanoString signature, generating continuous module-level measures of immune activity. This approach is consistent with established gene set scoring frameworks, including ssGSEA and GSVA, which summarize predefined gene sets into sample-level scores for downstream analysis (Barbie et al., 2009; Foroutan et al., 2018), and is analogous to module scoring methods used in transcriptomic tools such as Seurat (Stuart et al., 2019).

Associations between immune modules were assessed using Spearman’s rank correlation and visualized as hierarchical heatmaps (Euclidean distance, complete linkage), with non-significant correlations (p ≥ 0.05) masked. Analyses were performed in R (v4.6.0) using limma, Hmisc, and pheatmap.

### 2.5 Metabolomics and lipidomics

Untargeted metabolomics and lipidomics were performed on serum using an Agilent 1290 series ultra-high-performance liquid chromatography (UHPLC) system coupled to a Bruker Impact II quadrupole time-of-flight mass spectrometer (QTOF-MS). Samples were analyzed in two chromatographic modes for metabolomics: reverse-phase (RP) LC in positive electrospray ionization (ESI+) mode and hydrophilic interaction chromatography (HILIC) in negative ionization (ESI-) mode to maximize metabolome coverage. They were analyzed in RP positive and negative modes for lipidomics analysis.

#### 2.5.1 Metabolomics reverse-phase LC-MS (ESI+ mode)

Chromatographic separation was performed on a Waters Acquity BEH C18 column (1 mm × 100 mm, 1.7 µm particle size) maintained at 35 °C. The mobile phases consisted of water with 0.1% formic acid as solvent A and acetonitrile with 0.1% formic acid as solvent B. The gradient elution program was as follows: 5% B at 0 min, ramping to 25% B at 8 min, 70% B at 14 min, 95% B at 20 min, held at 95% B until 23 min, and then returned to 5% B at 23.1 min for column re-equilibration. The flow rate was 0.15 mL/min, and the injection volume was 5 µL.

#### 2.5.2 Metabolomics reverse-phase LC-MS (ESI- mode)

Chromatographic separation was performed on a Waters Acquity BEH C18 column (1 mm × 100 mm, 1.7 µm particle size) maintained at 35 °C. The mobile phases consisted of 95/5 water/acetonitrile with 10 mM ammonium acetate (pH = 9.8) as solvent A and 95/5 acetonitrile/water as solvent B. The gradient elution program was as follows: 5% B at 0 min, ramping to 25% B at 8 min, 70% B at 14 min, 95% B at 20 min, held at 95% B until 23 min, and then returned to 5% B at 23.1 min for column re-equilibration. The flow rate was 0.15 mL/min, and the injection volume was 5 µL.

#### 2.5.3 Metabolomics HILIC LC-MS (ESI+ mode)

Polar metabolites were separated using an Atlantis Premier z-HILIC column (2.1 mm × 150 mm, 2.5 µm particle size) maintained at 35 °C. The mobile phases consisted of 95/5 water/acetonitrile with 10 mM ammonium acetate (pH = 9.8) as solvent A and 95/5 acetonitrile/water as solvent B. The gradient started at 95% B, decreased to 50% B at 18 min, rapidly to 5% B at 18.1 min, held at 5% B until 20 min, then returned to 95% B at 21 min and maintained for column re-equilibration. The flow rate was 0.30 mL/min, and the injection volume was 4 µL.

#### 2.5.4 Metabolomics HILIC LC-MS (ESI- mode)

Polar metabolites were separated using an Atlantis Premier z-HILIC column (2.1 mm × 150 mm, 2.5 µm particle size) maintained at 35 °C. The mobile phases consisted of 95/5 water/acetonitrile with 10 mM ammonium acetate (pH = 9.8) as solvent A and 95/5 acetonitrile/water as solvent B. The gradient started at 95% B, decreased to 50% B at 18 min, rapidly to 5% B at 18.1 min, held at 5% B until 20 min, then returned to 95% B at 21 min and maintained for column re-equilibration. The flow rate was 0.30 mL/min, and the injection volume was 6 µL.

#### 2.5.5 Lipidomics reverse-phase LC-MS (ESI+ mode)

Chromatographic separation was performed on a Waters Acquity BEH C18 column (1 mm × 100 mm, 1.7 µm particle size) maintained at 35 °C. The mobile phases consisted of 40/60 water/acetonitrile with 2 mM ammonium formate (pH = 4.8) as solvent A and 90/10 isopropanol/acetonitrile as solvent B. The gradient elution program was as follows: 5% B at 0 min, ramping to 40% B at 8 min, 70% B at 14 min, 95% B at 20 min, held at 95% B until 23 min, and then returned to 5% B at 23.1 min for column re-equilibration. The flow rate was 0.10 mL/min, and the injection volume was 1 µL.

#### 2.5.6 Lipidomics reverse-phase LC-MS (ESI- mode)

Chromatographic separation was performed on a Waters Acquity BEH C18 column (1 mm × 100 mm, 1.7 µm particle size) maintained at 35 °C. The mobile phases consisted of 40/65 water/acetonitrile with 5 mM ammonium acetate (pH = 9.8) as solvent A and 90/10 isopropanol/acetonitrile as solvent B. The gradient elution program was as follows: 5% B at 0 min, ramping to 40% B at 8 min, 70% B at 14 min, 95% B at 20 min, held at 95% B until 23 min, and then returned to 5% B at 23.1 min for column re-equilibration. The flow rate was 0.10 mL/min, and the injection volume was 2 µL.

#### 2.5.7 Metabolomics mass spectrometry parameters

The mass spectrometer was operated in both positive and negative electrospray ionization modes. Samples were acquired in full scan mode whereas QCs were acquired in DDA mode. For reverse-phase positive mode, capillary voltage was set to 4500 V, with a drying gas temperature of 220 °C, gas flow of 7.0 L/min, and nebulizer pressure of 1.6 bar. Spectra were acquired over an m/z range of 70–1500, with a spectral acquisition rate of 2.00 Hz. For HILIC positive mode, capillary voltage was set to 4500 V, with a drying gas temperature of 250 °C, gas flow of 9.0 L/min, and nebulizer pressure of 2.5 bar. Spectra were acquired over an m/z range of 70–1500, with a spectral acquisition rate of 8.00 Hz. For reverse-phase negative mode, capillary voltage was set to 3600 V, with a drying gas temperature of 220 °C, gas flow of 7.0 L/min, and nebulizer pressure of 1.6 bar. Spectra were acquired over an m/z range of 70–1500, with a spectral acquisition rate of 8.00 Hz. For HILIC negative mode, capillary voltage was set to 3600 V, with a drying gas temperature of 250 °C, gas flow of 9.0 L/min, and nebulizer pressure of 2.5 bar. Spectra were acquired over an m/z range of 70–1500, with a spectral acquisition rate of 2.00 Hz.

#### 2.5.8 Lipidomics mass spectrometry parameters

The mass spectrometer was operated in both positive and negative electrospray ionization modes. Samples and QCs were all acquired in DDA mode. For positive mode, capillary voltage was set to 4500 V, with a drying gas temperature of 220 °C, gas flow of 6.0 L/min, and nebulizer pressure of 1.0 bar. Spectra were acquired over an m/z range of 70–2500, with a spectral acquisition rate of 8.00 Hz. For negative mode, capillary voltage was set to 3600 V, with a drying gas temperature of 220 °C, gas flow of 6.0 L/min, and nebulizer pressure of 1.0 bar. Spectra were acquired over an m/z range of 70–2500, with a spectral acquisition rate of 8.00 Hz.

#### 2.5.9 Data processing and metabolite annotation

Metabolic features were extracted using the Agilent Mass Profiler. Data were log-transformed and auto-scaled (mean-centered, and scaled to unit variance) using MetaboAnalystR (v6.0) (Pang et al., 2024). A total of 856 metabolic features differed between cases and controls based on an unpaired two-tailed t-test (p < 0.05); no multiple testing correction was applied at this exploratory stage. These nominally significant features at the post-infection timepoint underwent MS/MS-based identification against an in-house library and public databases (METLIN, HMDB), resulting in 45 metabolites being annotated, of which 24 were uniquely identified (**Supplementary Table 2**). Annotated metabolites were log-transformed, auto-scaled, and zero values were imputed as one-fifth of the smallest observed value.

### 2.6 Statistical analyses

To explore global multivariate patterns and assess separation between LC cases and controls, principal component analysis (PCA) and permutational multivariate analysis of variance (PERMANOVA), implemented using the vegan package in R, were performed on the complete gene expression and metabolomic datasets. PCA, an unsupervised dimensionality reduction technique, summarizes variation across multiple correlated features into a smaller number of principal components, facilitating visualization of the overall data structure and potential clustering of samples. PERMANOVA was used to test whether the multivariate centroids of LC cases and controls differed in feature space, providing an objective statistical assessment of group separation (A new method for non‐parametric multivariate analysis of variance - Anderson - Austral Ecology - Wiley Online Library, 2021). To ensure that observed differences reflected true separation between groups rather than unequal within-group variability, a *post hoc* permutational analysis of multivariate dispersion (PERMDISP) was also performed. PERMDISP evaluates the homogeneity of multivariate dispersions, with p-values > 0.05 indicating that group differences are more likely attributable to separation of centroids than to differences in within-group dispersion (Anderson, 2006).

Differential gene expression analysis between LC cases and controls was performed using the limma package (v3.58.1) in R (v4.5.0). Limma is well suited for small sample sizes because its empirical Bayes framework moderates gene-wise variance estimates, reducing the influence of outliers and improving statistical power (Ritchie et al., 2015). Top genes distinguishing LC cases from controls were identified using a Benjamini–Hochberg (BH) false discovery rate (FDR) < 0.10 and a fold change (FC) > 1.5. Because limma reports log2 fold change values, this corresponded in practice to an absolute threshold of |log2FC| > 0.585, which is equivalent to FC > 1.5. This FDR threshold was selected because the NanoString CodeSet comprises a preselected, biologically relevant panel of genes with correlated expression, which may violate the independence assumptions underlying more conservative multiple-testing corrections and could otherwise increase false negatives and obscure meaningful signals. Thus, the use of FDR < 0.10 balanced control of false positives with sufficient sensitivity to detect relevant differences in this focused, hypothesis-driven dataset. Differential abundance analysis of metabolites was also performed using limma, applying a conventional BH FDR threshold of < 0.05.

Given the small sample sizes in both analyses, transcriptomics (LC cases, n = 8; controls, n = 29) and metabolomics (LC cases, n = 6; controls, n = 6), statistical power was limited, reducing the ability to detect modest effect sizes in high-dimensional data. To complement FDR-adjusted p-values, Cohen’s d effect sizes were calculated for each gene and metabolite to quantify the magnitude of between-group differences independent of sample size. Larger absolute Cohen’s d values indicate greater separation between group means relative to within-group variability.

Longitudinal changes in metabolite abundance from pre- to post-infection were analyzed using two-tailed paired t-tests and linear mixed-effects models (LMM) (lmer, R). Paired t-tests were used to evaluate within-subject differences over time, providing a direct measure of metabolite changes associated with infection. LMMs were then applied to account for both fixed effects (time, group, and their interaction) and random effects (subject-level variability), allowing for robust inference despite repeated measures and potential imbalance across individuals. This approach provides a more comprehensive assessment of whether metabolite trajectories differed between LC cases and controls over time (Bates et al., 2015). P-values from both analyses were adjusted for multiple comparisons using the BH FDR method.

### 2.7 Transcriptomics and metabolomics network integration and pathway analysis

To mitigate the limitations associated with the small sample size, we employed a network-based analysis integrating transcriptomic and metabolomic data to generate a comprehensive protein–protein interaction map and enable pathway-level analyses, which are generally more reliable than single-feature approaches. Significant annotated metabolites were mapped via MetaCyc to their corresponding enzyme-coding genes using Metabridge (Al-Aly et al., 2024) and integrated with differentially expressed genes in NetworkAnalyst (v3.0) (Greenhalgh et al., 2022) to construct a zero-order protein–protein interaction network. Because metabolite annotations in untargeted metabolomics are inherently limited, only a subset of detected features could be confidently annotated and mapped to genes. This reflects a recognized limitation of untargeted metabolomics, in which many detected features remain unannotated due to incomplete spectral reference libraries and the absence of a template-based identification framework (Lee et al., 2025). Accordingly, the integrated network was used as an exploratory, hypothesis-generating approach rather than a comprehensive representation of transcript–metabolite interactions. Enriched pathways from the Kyoto Encyclopedia of Genes and Genomes (KEGG) (Kanehisa and Goto, 2000; Kanehisa, 2019; Kanehisa et al., 2025) were identified using over-representation analysis (ORA), which tests whether the number of observed hits (genes or metabolites) in a given pathway exceeds that expected by chance under a hypergeometric distribution, relative to the total background set of genes or metabolites. Pathways with an FDR-adjusted p < 0.05 were considered significantly enriched.

### 2.8 Cross-cohort concordance and replication analysis

Limma was used to assess replication of differential gene expression and metabolite abundance across two independent transcriptomic cohorts and one metabolomic cohort. Cross-cohort consistency was evaluated by fold-change directionality, and Spearman correlations were computed between discovery and external cohort gene and metabolite signatures. Analyses were performed in R (v4.6.0).

## 3 Results

### 3.1 Participants

**Figure 1:**
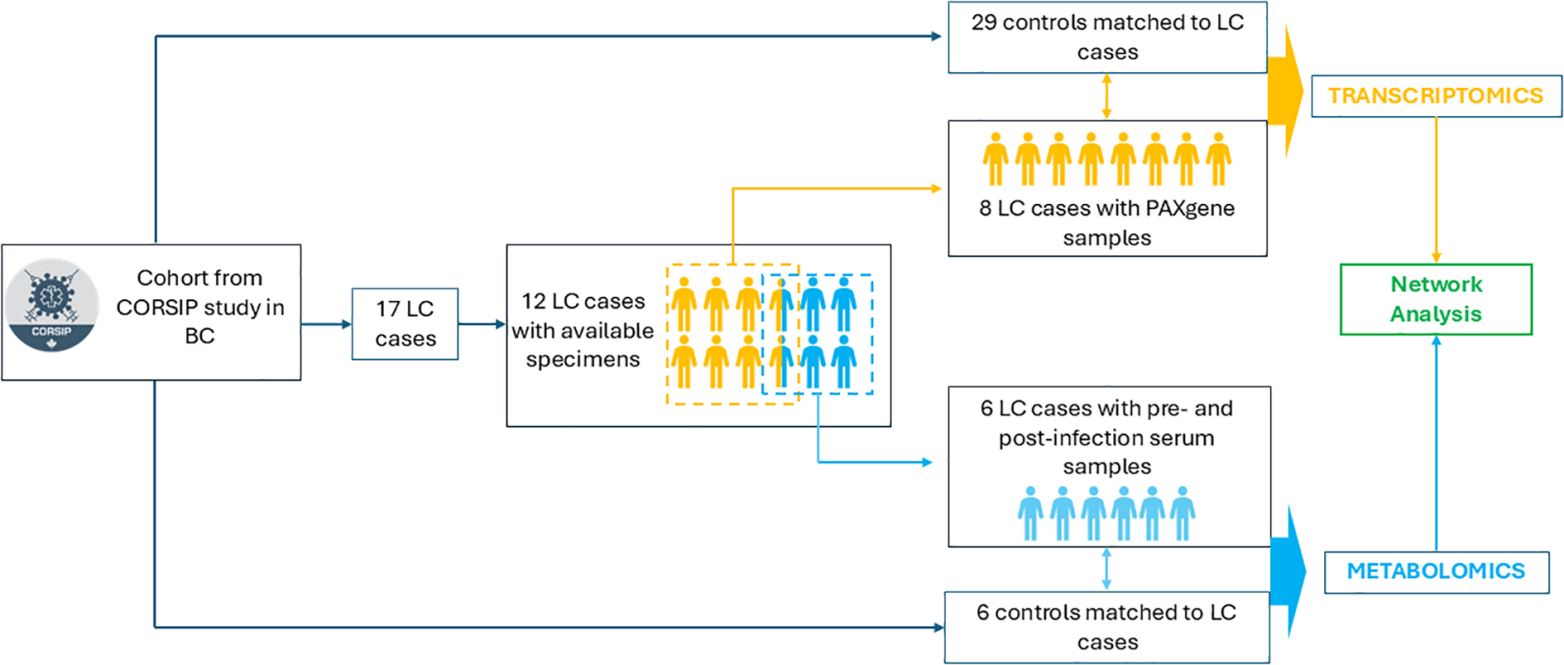
Study design and cohort overview. Transcriptomic and metabolomic profiles were compared between LC cases and matched controls in the BC CORSIP paramedic cohort. Eight cases were analyzed for transcriptomics and six for metabolomics, with two cases contributing to both assays. Controls were matched by age, sex, and sample timing.

We included a total of 12 LC cases and 35 controls (individuals who had COVID-19 but recovered without any persistent symptoms) from the CORSIP study (Grunau B, 2020). Of these, 8 LC cases and 29 controls underwent transcriptomic analysis based on PAXgene sample availability, while 6 LC cases and 6 controls underwent metabolomic analysis based on paired pre- and post-infection serum sample availability. Two LC participants contributed to both assays, providing both PAXgene (transcriptomics) and serum (metabolomics) samples (**Figure 1**).

The most common symptoms among LC participants were fatigue (67%) and brain fog (58%). The median age was 43.5 years in LC cases and 37.0 years in controls. Women comprised 67% of LC cases and 40% of controls. The median body mass index (BMI) was 32.8 kg/m^2^ in cases and 29.4 kg/m^2^ in controls. None of the participants were hospitalized during their acute infection. Most infections occurred during the Omicron wave (75% of LC cases and 96% of controls), and similar proportions of participants had received their first SARS-CoV-2 vaccine dose prior to infection. These potential confounding variables were balanced between LC cases and controls, as assessed using the Mann–Whitney U test for continuous variables and Fisher’s exact test for categorical variables (**Table 1**).

### 3.2 Transcriptomics

**Figure 2:**
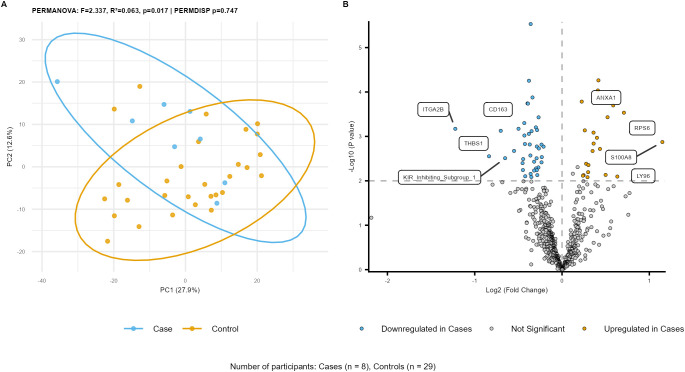
Transcriptomics profiling. **(A)** PCA of whole gene expression shows separation between LC cases and controls (PERMANOVA: R^2^ = 0.063, p = 0.016; PERMDISP: p = 0.747). **(B)** Volcano plot of 63 differentially expressed genes (FDR < 0.10), with genes labeled if |FC| > 1.5.

PCA, a dimensionality reduction technique that summarizes global variation in high-dimensional data, revealed a separation between LC cases and controls when applied to the global gene expression data (**Figure 2A**). PERMANOVA, which tests whether group centroids differ in multivariate space, identified a statistically significant difference in multivariate profiles between LC cases and controls (F = 2.337, R^2^ = 0.063, p = 0.016), although the effect size was modest, with group status explaining 6.3% of the total variance. PERMDISP confirmed that this difference was not driven by unequal within-group dispersion (p = 0.747), supporting the interpretation that the observed separation reflects true differences in group centroids. Given the modest sample size, these findings should be interpreted cautiously, as limited statistical power may have reduced the ability to detect additional transcriptomic alterations. Nevertheless, the convergence of multivariate, differential expression, and pathway-level signals suggests that the identified immune-related patterns are unlikely to be attributable to random variation alone.

Differential expression analysis with limma identified 63 genes that distinguished LC cases from controls, with p-values adjusted for multiple comparisons using the BH-FDR < 0.10. Cohen’s *d* effect size coefficients for these genes were >1, suggesting relatively large effects (**Supplementary Table 3**). Of these 63 differentially expressed genes (DEGs), eight exhibited a fold change of |FC| > 1.5, highlighting genes with potentially greater effect sizes and biological relevance (**Figure 2B**). These included markers of platelet activation (*ITGA2B, THBS1*), innate immunity (*S100A8, LY96, ANXA1, CD163)*, NK-cell regulation (*KIR2DL1*), and protein translation (*RPS6*) (The Gene Ontology Consortium, 2015).

#### 3.2.1 Immune transcriptional profiling

Given that both *S100A8* (a monocyte-associated gene) and *KIR2DL1* (an NK cell–related inhibitory receptor) were significantly differentially expressed and exhibited an absolute fold change >1.5, they were selected for immune transcriptional profiling. Because *S100A8* was upregulated in LC cases, we hypothesized that it may reflect persistent myeloid activation. Correlation analyses using NanoString immune modules (**Supplementary Table 1**) showed a strong positive association between *S100A8* and the neutrophil signature (ρ = 0.68, p < 0.001), but no significant association with the monocyte/macrophage module (ρ = −0.27, p = 0.107), cytotoxic score (ρ = −0.07, p = 0.673), or NK function (ρ = −0.15, p = 0.365). These findings suggest that *S100A8* is more closely linked to neutrophil-related inflammation than to broader myeloid or cytotoxic activity.

In contrast, *KIR2DL1* showed significant positive correlations with NK-cell identity (ρ = 0.81, p = 1.0 × 10_-7_) and NK functional activity (ρ = 0.58, p < 0.001), and negative correlations with *S100A8* (ρ = −0.48, p = 0.0032) and the neutrophil signature (ρ = −0.39, p = 0.017), suggesting divergent regulation of NK- and neutrophil-associated inflammatory programs. Given the targeted nature of the NanoString panel and the limited sample size, these findings should be considered exploratory but may reflect distinct immune programs associated with NK regulatory signaling and neutrophil-driven inflammation in LC.

**Figure 3:**
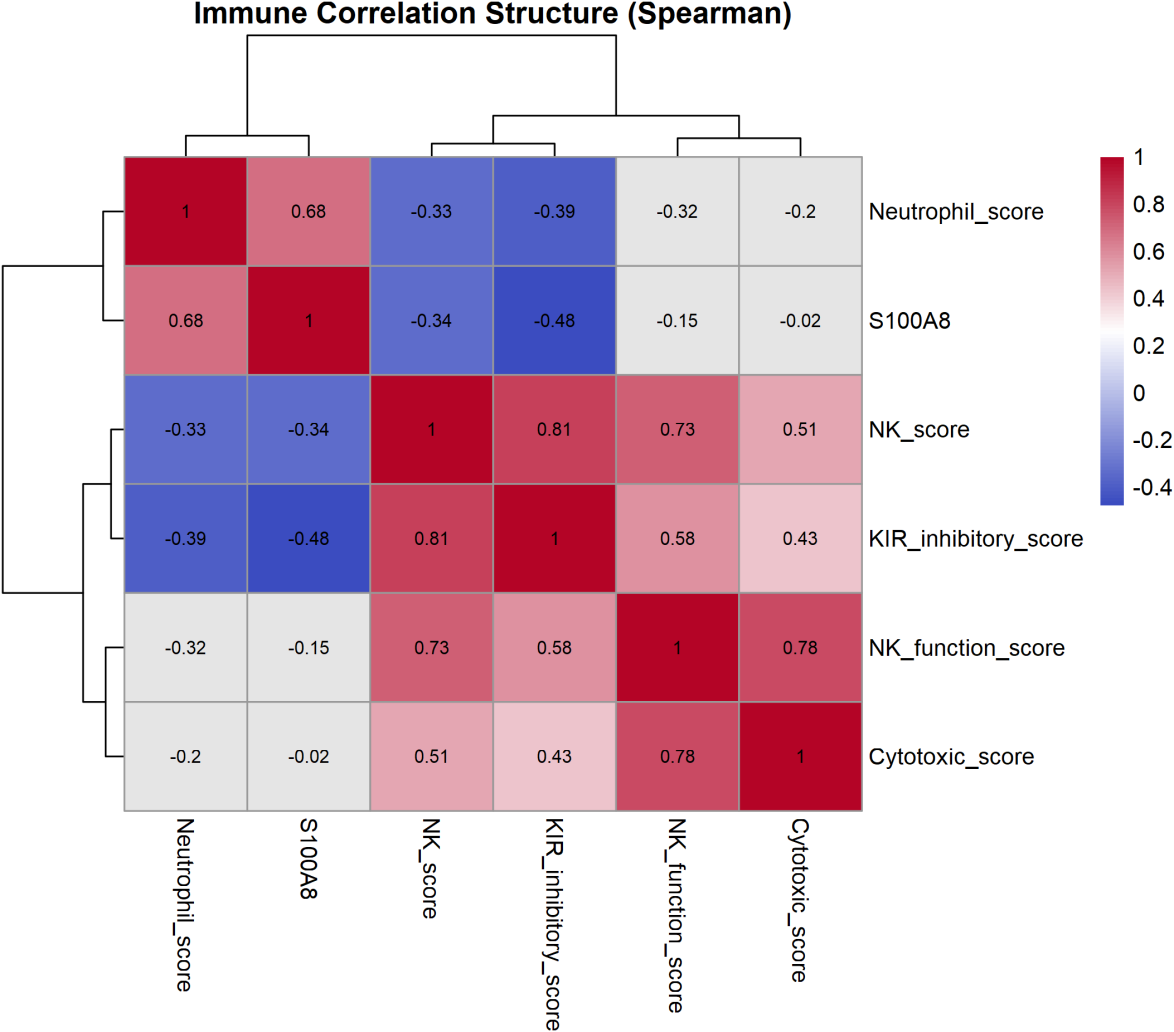
Correlation matrix across NK regulatory, inflammatory, and cytotoxic NanoString modules, *S100A8* and *KIR2DL1* expression. Heatmap of Spearman correlation coefficients (ρ) between predefined immune features, including *S100A8* expression and *KIR2DL1* signaling. Non-significant correlations (p ≥ 0.05) were masked for visualization. Correlation values range from −1 (strong negative correlation) to +1 (strong positive correlation). Hierarchical clustering was performed using Euclidean distance and complete linkage.

To visualize global immune structure, we constructed a Spearman correlation matrix including NK-cell score, NK functional score, cytotoxicity score, neutrophil signature, and *S100A8* and *KIR2DL1* expression. Hierarchical clustering identified two major modules: an NK-associated module (NK-cell score, NK function, *KIR2DL1*) and a neutrophil/inflammatory module (*S100A8*, neutrophil signature). Cross-module correlations were weak or negative, consistent with compartmentalized immune regulation in LC in this pilot study (**Figure 3**).

### 3.3 Metabolomics

**Figure 4:**
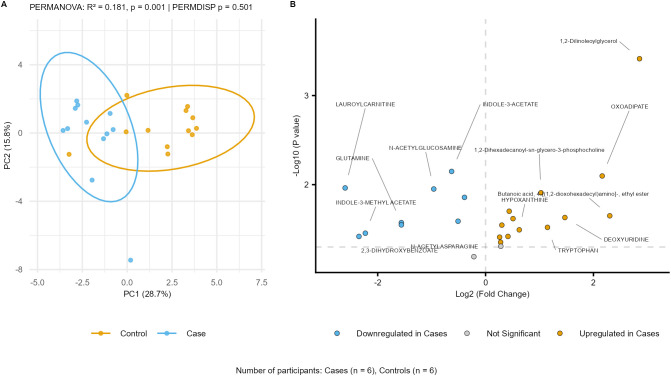
Metabolomics profiling. **(A)** PCA of post-infection annotated metabolites reveals group separation (PERMANOVA: R^2^ = 0.181, p = 0.001; PERMDISP: p = 0.498). **(B)** Volcano plot of 22 differentially abundant metabolites (FDR < 0.05), with metabolites labeled if |FC| > 1.5.

PCA of all annotated metabolites at the post-infection timepoint revealed separation between LC cases and controls. PERMANOVA indicated that group centroids differed significantly (R^2^ = 0.181, p = 0.001), while PERMDISP confirmed similar within-group dispersions (p = 0.498), supporting that the separation reflects true group differences (**Figure 4A**). Even though these findings should be interpreted cautiously, given that the sample size may have limited the detection of additional metabolomic alterations and increased susceptibility to false negatives, the observed group separation and the identification of biologically relevant metabolites support the presence of measurable metabolic differences associated with LC.

Metabolic features differentially abundant at the post-infection timepoint in LC cases compared to controls were identified using a two-tailed t-test (p < 0.05). A total of 856 features were detected, of which 45 metabolites could be annotated, and 24 metabolites had unique annotations, including lipids, acylcarnitines, amino acids, nucleotide derivatives, and N-acetylglucosamine (**Supplementary Table 2**).

Furthermore, limma analysis identified 22 metabolites that discriminated LC cases from controls (FDR < 0.05), 14 of which exhibited a |FC| > 1.5. These included 1,2-dilinoleoylglycerol, indole-3-acetate, oxoadipate, lauroylcarnitine, N-acetylglucosamine, 1,2-dihexadecanoyl-sn-glycero-3-phosphocholine, butanoic acid, 4-[(1,2-dioxohexadecyl)amino]-, ethyl ester, deoxyuridine, glutamine, N-acetylasparagine, tryptophan, hypoxanthine, indole-3-methyl acetate, and 2,3-dihydroxybenzoate (**Figure 4B**). These metabolites also exhibited Cohen’s d coefficients >1, suggesting relatively large effect sizes and potential biological relevance (**Supplementary Table 2**).

**Figure 5:**
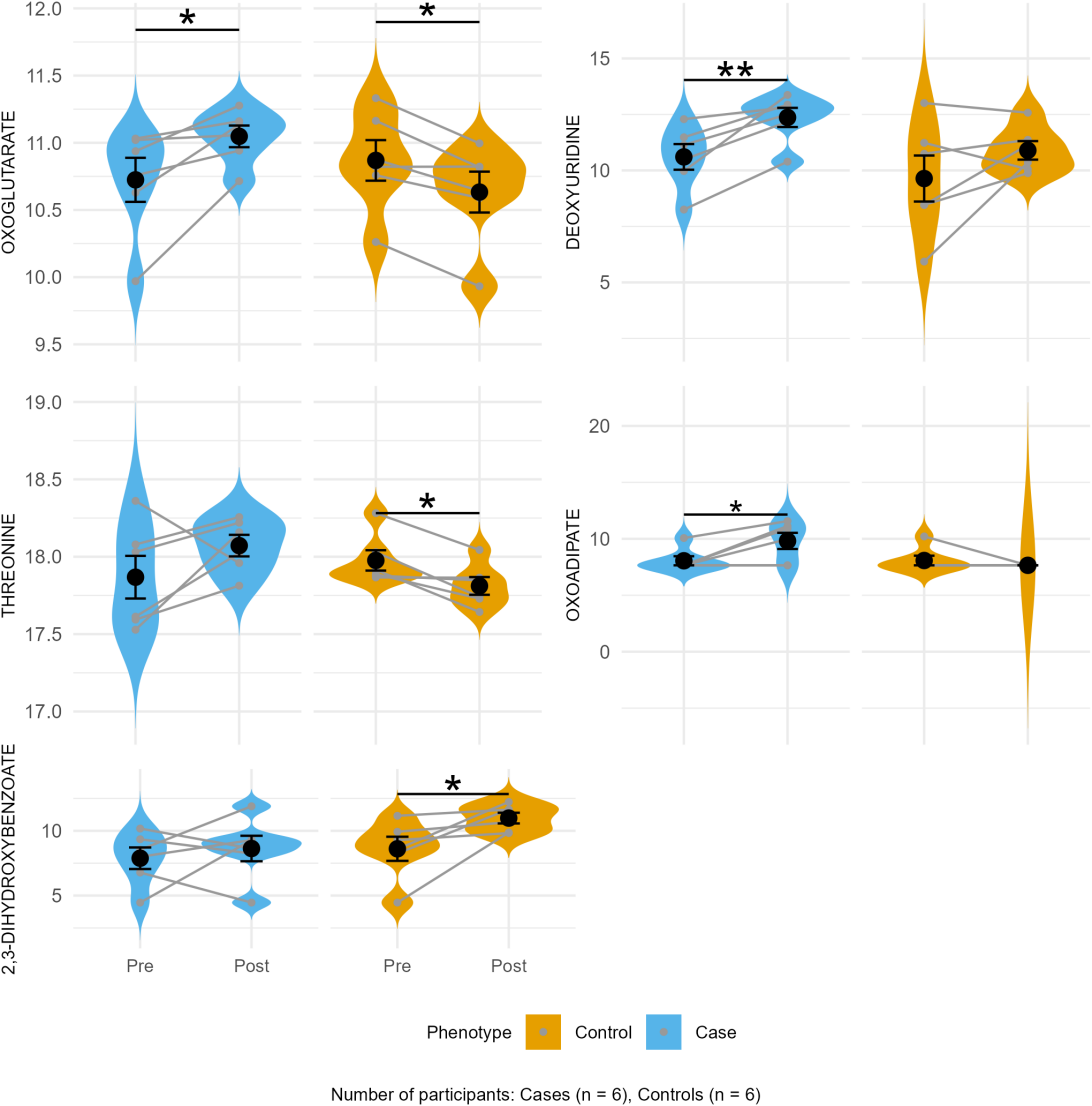
Longitudinal metabolomic changes. Oxoglutarate exhibited a significant time-by-LC interaction (linear mixed-effects model: *p* = 9.7 × 10⁻⁴, FDR = 0.023), driven by an increase in LC cases and a decrease in controls over time. Paired t-tests identified nominal (unadjusted) within-group changes for deoxyuridine, oxoglutarate, and oxoadipate in LC cases, and for oxoglutarate, threonine, and 2,3-dihydroxybenzoate in controls; none remained significant after FDR correction. Error bars represent standard error of the mean (SEM). In the oxoadipate panel, overlapping trajectories may appear indistinguishable due to identical or near-identical values across participants.

In the longitudinal analysis, metabolite changes from pre- to post-infection were evaluated using two-tailed paired t-tests to assess within-subject differences, and linear mixed-effects models (LMM) to account for fixed effects (time, group, and their interaction) and random effects (subject-level variability). Among metabolites, oxoglutarate exhibited a significantly distinct trajectory, increasing in LC cases but decreasing in controls, with a robust time-by-LC interaction in the LMM (p = 0.00097, FDR = 0.023; **Figure 5**). Oxoadipate and threonine also showed nominally significant time-by-group interactions (p = 0.02 and p = 0.03, respectively), though these did not remain significant after FDR correction (**Supplementary Table 4**).

Paired t-test analyses further revealed nominal pre- to post-infection changes in both groups. In LC cases, deoxyuridine (p = 0.006), oxoglutarate (p = 0.030), and oxoadipate (p = 0.038) increased following infection. In controls, oxoglutarate (p = 0.008) and threonine (p = 0.022) decreased, while 2,3-dihydroxybenzoate increased (p = 0.038). None of these associations remained significant after FDR adjustment (**Figure 5**).

### 3.4 Transcriptomics and metabolomics network integration and pathway analysis

**Figure 6:**
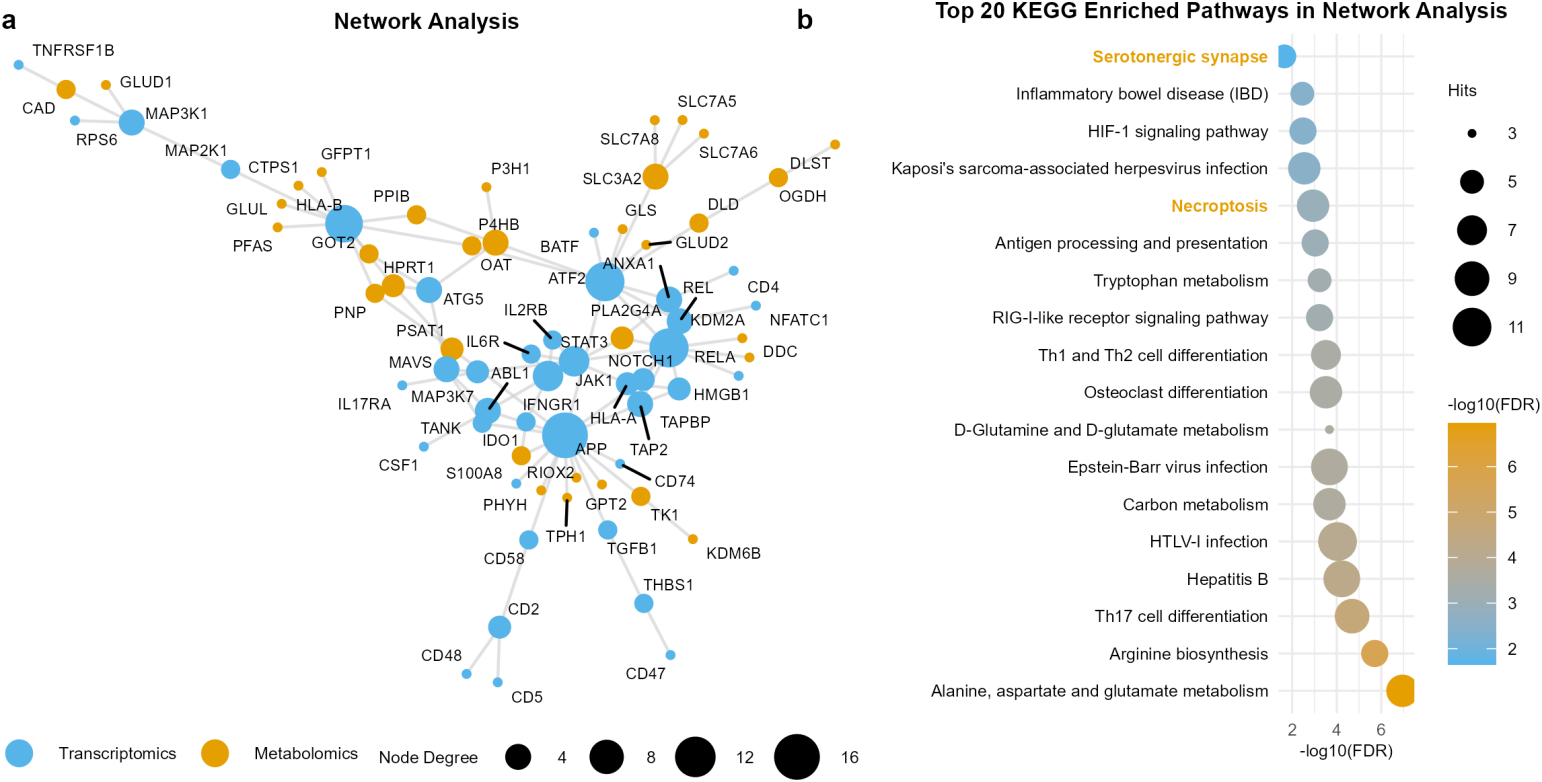
Integrated network analysis. **(A)** Protein–protein interaction network highlighting central nodes (APP, ATF2, RELA, HLA-B). **(B)** KEGG pathway enrichment (FDR < 0.05) identified pathways related to metabolism, immunity, viral/infectious responses, and inflammation/signaling. Necroptosis and serotonergic synapse were enriched in both transcriptomic and metabolomic datasets.

To account for the small sample size, we applied an integrative multi-omics framework combining transcriptomic, metabolomic, and network-based analyses, focusing on pathway-level rather than single-feature approaches. The 63 DEGs from the transcriptomic analysis were integrated with metabolites showing significantly altered abundance in LC cases versus controls at the post-infection timepoint. For integration, metabolites were mapped to their corresponding enzyme-coding genes within a protein–protein interaction network. Core nodes of this network, identified as hubs with the highest degrees (i.e., the largest number of direct interactions), included APP, RELA, ATF2, and HLA-B (degrees 16, 11, 11, and 10, respectively; **Figure 6A**).

KEGG pathway enrichment analysis (FDR < 0.05) highlighted pathways related to metabolism (amino acid, central carbon, and nitrogen metabolism), immunity (Th1/Th2/Th17, antigen presentation, NK cell activity, T/B cell signaling), viral and infectious responses, and inflammation/signaling (HIF-1, PI3K-Akt, MAPK, TNF, IL-17) (Kanehisa and Goto, 2000) (**Figure 6B**; **Supplementary Table 5**). Notably, necroptosis and serotonergic synapse pathways were significantly enriched in both the transcriptomic and metabolomic datasets (FDR < 0.05).

### 3.5 Cross-cohort concordance and replication analysis

Cross-cohort validation was performed using independent transcriptomic and metabolomic datasets to assess the reproducibility of the DEGs, metabolites, and pathway-level signals identified in our cohort, with the aim of addressing the limitation of a small sample size.

In Transcriptomic Cohort A (GSE224615), all 63 DEGs were represented, and 54.0% showed concordant direction of change, although fold-change correlations were not significant (Spearman’s ρ = 0.05, p = 0.68; **Supplementary Table 6**). In Transcriptomic Cohort B (GSE275334), 24 DEGs overlapped with the external dataset, of which 45.8% showed concordant directionality, again without significant fold-change correlation (Spearman’s ρ = −0.12, p = 0.57; **Supplementary Table 6**).

In the external metabolomics cohort, 7 of the 24 significant metabolites were represented, and 85.7% showed concordant direction of change, although fold-change correlations were not significant (Spearman’s ρ = 0.53, p = 0.23; **Supplementary Table 7**).

None of the DEGs or metabolites reached FDR significance in the external cohorts. This limited replication likely reflects major methodological and cohort differences across studies, including profiling platforms (NanoString vs RNA-seq), biospecimen types (whole blood vs PBMCs; serum vs plasma), hospitalization status, and overall cohort heterogeneity.

Despite the absence of statistical replication, several immune-related genes, including *CD4, IL6R, CD163, LAMP1, NFATC1*, and *HLA-E*, showed consistent directionality across datasets. Similarly, multiple features contributing to the necroptosis and serotonergic synapse pathways identified through integrative analyses, including glutamine, oxoglutarate, tryptophan, *STAT3, HMGB1, JAK1, IFNGR1*, and *APP*, also showed concordant directionality (**Supplementary Table 8**).

Overall, these findings provide limited gene-level replication but modest pathway-level concordance across independent datasets, supporting the exploratory hypothesis of immune-metabolic dysregulation in LC while underscoring the need for validation in larger, better-matched cohorts.

## 4 Discussion

To our knowledge, this is the first exploratory study integrating transcriptomics and metabolomics in non-hospitalized healthcare worker populations with LC. Given the small sample size and substantial heterogeneity of LC, these findings should be interpreted cautiously. A recent narrative review synthesizing 56 studies in healthcare workers reported marked variability in symptom definitions, prevalence, and risk factors, while underlying mechanisms remain incompletely understood (Dempsey et al., 2024). This heterogeneity underscores the value of integrative molecular profiling approaches in well-defined subpopulations.

We examined the transcriptomic and metabolomic profiles of non-hospitalized healthcare workers with LC, a population underrepresented in hospital-based studies despite their high infection risk and ~40% prevalence of persistent symptoms after 22 weeks of follow-up (O’Mahoney et al., 2023). The relatively small sample size reflects the challenges of studying a well-characterized occupational cohort with longitudinal biospecimen collection, particularly given the requirement for paired pre-infection samples, which remain uncommon in LC research. In our cohort, fatigue (67%) and brain fog (58%) were the most common symptoms, with a median age of 43.5 years and a predominance of women (67%). These characteristics mirror previous reports describing similar symptom profiles and female predominance in both healthcare workers and non-hospitalized LC populations (Bell et al., 2021; O’Mahoney et al., 2023). This consistency may support the representativeness of our cohort within similar non-hospitalized healthcare workers populations marked by fatigue and cognitive dysfunction, particularly among women, potentially reflecting sex-related differences in immune regulation, hormonal signaling, or occupational exposure.

Transcriptomic analysis revealed 63 dysregulated genes in LC cases. Notably, *S100A8* and *LY96*, genes distinguishing LC cases from controls, together with *HMGB1* and *RELA*, converge on a neutrophil-driven inflammatory axis. These findings align with reports describing myeloid activation and Toll-like receptor 4 (TLR4) engagement during acute infection and post-acute inflammatory states of SARS-CoV-2 infection (Yao et al., 2022; Tsukalov et al., 2024), as well as elevated circulating S100A8, S100A9 and HMGB1 proteins observed in severe COVID-19 and in long COVID (Chen et al., 2020; Springe et al., 2026). *S100A8* and *S100A9* encode calprotectin, an amplifier of TLR4 signaling, while *LY96* encodes MD-2, a TLR4 co-receptor required for ligand recognition, that enhances responsiveness to inflammatory stimuli. *RELA*, a central node in our network analysis, encodes the NF-κB p65 subunit, a major mediator of cytokine transcription. Together, these molecules may sustain TLR4-mediated inflammatory signaling, potentially triggered by HMGB1, a damage-associated molecular pattern associated with neutrophil activation and tissue injury signaling (Oliveira et al., 2024; Jung et al., 2025).

In the immune profiling analyses using NanoString immune modules. *S100A8* showed a strong positive association with the neutrophil signature, but no significant associations with monocyte/macrophage, cytotoxic, or NK functional modules, suggesting a more specific relationship with neutrophil-driven inflammation rather than generalized myeloid activation. This pattern is consistent with a preclinical post-acute SARS-CoV-2 model in which neutrophils exhibited a sustained pro-inflammatory and pro-fibrotic transcriptional program marked by persistent upregulation of S100A8/S100A9 across multiple tissues. In the same study, pharmacologic inhibition of S100A8/A9 reduced LC incidence (Yu et al., 2025).

In LC clinical studies, increased neutrophil activity has also been reported, including enrichment of neutrophil degranulation pathways (Di Ciaula et al., 2024), and neutrophil-driven immune activation signatures in non-hospitalized LC cases with neuropsychiatric symptoms (Lin et al., 2024). Consistent with these observations, *ARG1*, also dysregulated in our study, is expressed in neutrophils and myeloid regulatory populations and is linked to post-viral immune dysregulation, further supporting persistent neutrophil-driven inflammation in LC (Derakhshani et al., 2021).

In contrast, *KIR2DL1* correlated positively with NK-cell identity and functional activity, and negatively with *S100A8* and neutrophil signatures, suggesting divergent regulation between NK-associated and neutrophil-associated immune programs. Hierarchical clustering further supported this separation, identifying two major modules: an NK-associated module (NK-cell score, NK function, *KIR2DL1*) and a neutrophil/inflammatory module (*S100A8*, neutrophil signature), with weak or negative cross-module correlations suggesting compartmentalized immune organization in this exploratory cohort. This observation aligns with previous reports of NK-cell dysregulation and altered cytotoxic phenotypes in LC, including reduced NK-cell frequencies and lower proportions of NK cells expressing *KIR2DL1* and related receptors in severe LC, a phenotype associated with impaired clearance of virus-infected cells. These alterations were particularly evident in individuals with neurocognitive, gastrointestinal, and fatigue-related symptoms, suggesting that disrupted NK-cell regulatory and cytotoxic programs may contribute to persistent symptomatology (Tsao et al., 2025).

Together, the inverse relationship between *KIR2DL1*/NK modules and *S100A8*-neutrophil signatures may reflect differential regulation between cytotoxic NK-associated programs and persistent neutrophil-driven inflammatory pathways. Although exploratory, these findings raise the possibility that LC immune heterogeneity involves partially compartmentalized innate immune states characterized by either sustained neutrophil-associated inflammation or relatively preserved NK-associated regulatory activity. However, given the targeted nature of the NanoString platform and limited sample size, these findings should be interpreted as hypothesis-generating.

Metabolomic profiling revealed alterations in energy and nucleotide metabolism. Tryptophan, asparagine, oxoglutarate, deoxyuridine, ureidopropionate, and thiamine monophosphate were among the metabolites associated with separation between LC cases and controls. LC cases exhibited altered tryptophan levels, consistent with previous studies (Wong et al., 2023). Such alterations may reflect dysregulated tryptophan–kynurenine pathway activity potentially involving indoleamine 2,3-dioxygenase induction, and gut dysbiosis (Yao et al., [[NoYear]]) and may contribute to immune–neuroendocrine imbalance. Although a meta-analysis reported decreased circulating tryptophan levels in LC (Almulla et al., 2024), differences between serum, and plasma concentrations have been described and may explain the higher serum levels observed in our cohort (Almulla et al., 2022).

We also observed elevated asparagine levels in LC cases, consistent with reports that asparagine and aspartate increase during SARS-CoV-2 infection through activation of the integrated stress response (Zhang et al., 2021). This elevation may reflect persistent metabolic stress and dysregulated amino acid homeostasis. Deoxyuridine levels were also increased in LC cases, as previously described during acute infection, which may indicate impaired pyrimidine metabolism potentially driven by altered nucleotide turnover or viral interference with host replication pathways (Narayanan et al., 2024). Similarly, ureidopropionate, a pyrimidine degradation product, was elevated and has been associated with infection outcomes, potentially reflecting activation of salvage pathways (Roberts et al., 2022).

Longitudinal analysis showed that oxoglutarate, a tricarboxylic acid (TCA) cycle intermediate, followed divergent trajectories between groups, increasing from pre- to post-infection, rising in LC cases while decreasing in controls. This bidirectional pattern may reflect altered TCA cycle activity and systemic metabolic stress rather than a transient acute-phase response. Elevated oxoglutarate has also been reported in severe COVID-19, suggesting that TCA cycle dysregulation may contribute to susceptibility, disease severity or recovery trajectories. SARS-CoV-2 is known to alter host cellular metabolism and increase ATP demand to support viral replication. Additionally, oxoglutarate levels have been reported to correlate with cytokine expression, potentially linking metabolic alterations to immune activation (Jia et al., 2021).

Integration of transcriptomic and metabolomic data through a protein–protein interaction network was performed to generate hypotheses about pathways potentially involved in LC biology. Metabolite mapping is limited by the inherent sparsity of annotations; consequently, only a subset of detected features could be confidently mapped to genes. This reflects a well-recognized limitation of untargeted metabolomics, in which many detected features remain unannotated due to incomplete reference libraries and the absence of a template-based identification framework (Novoa-del-Toro and Witting, 2024; Lee et al., 2025; Xu and Zhu, 2025). Accordingly, the integrated network should be interpreted as an exploratory, hypothesis-generating tool rather than a comprehensive representation of transcript–metabolite interactions. Within these constraints, *APP, ATF2, RELA*, and *HLA-B* emerged as central nodes. Dysregulation of *APP* has been associated with neuroinflammation and cognitive symptoms such as brain fog (Patel et al., 2022). SARS-CoV-2 infection has been shown to upregulate *APP* expression and perturb amyloid processing, potentially linking viral persistence to neurodegenerative pathways (Liu et al., 2023). Downregulation of HLA-B, a key component of antigen presentation and NK-cell regulation, may indicate impaired immune recognition, a mechanism previously implicated in chronic post-viral fatigue syndromes (Peppercorn et al., 2023).

Necroptosis and serotonergic synapse pathways were uniquely enriched through integrative analysis of transcriptomic and metabolomic datasets. Necroptosis, a regulated form of programmed cell death implicated in acute COVID-19, may contribute to sustained inflammatory signaling and tissue injury in LC (Kang and Wang, 2022). While neutrophil activation has been proposed to amplify inflammatory damage and vascular dysfunction through cell death–associated mechanisms, including in acute COVID-19 autopsy and single-cell studies, direct evidence of persistent necroptotic activity months after infection remains limited (Schweizer et al., 2021; Price et al., 2022; Poyatos et al., 2024).

Together, these observations may reflect disruption of the tryptophan–serotonin metabolic axis, potentially linking systemic metabolic dysregulation to neurocognitive symptoms in LC. Recent mechanistic studies support this hypothesis, suggesting that LC-associated cognitive impairment may involve blood–brain barrier disruption, immunovascular activation, and sustained systemic inflammation in individuals reporting brain fog (Greene et al., 2024). Reduced serotonergic signaling, potentially resulting from altered tryptophan metabolism, may contribute to fatigue, mood disturbances, and cognitive dysfunction commonly observed in LC (Wong et al., 2023).

Cross-cohort validation using independent transcriptomic and metabolomic datasets demonstrated limited replication of individual DEGs and metabolites. In Transcriptomic Cohort A (GSE224615), 54.0% of DEGs showed concordant directionality, while Cohort B (GSE275334) showed 45.8% concordance, without significant fold-change correlations. In the metabolomics dataset, 85.7% of overlapping metabolites showed consistent directional changes, although correlations did not reach statistical significance. None of the features achieved FDR significance in external datasets, which is expected given the limited availability of external cohorts closely matching our study design (whole-blood samples, non-hospitalized LC participants, and NanoString PanCancer Immune Profiling). We therefore selected the most comparable publicly available datasets, although substantial heterogeneity remained across transcriptomic platforms (NanoString vs RNA-seq), biospecimen types (whole blood vs PBMCs; serum vs plasma), and cohort composition (non-hospitalized vs mixed-severity populations). In addition, limited gene-level reproducibility across LC cohorts is well documented and reflects the biological and technical heterogeneity inherent to post-viral syndromes. Systematic multi-omics reviews of long COVID highlight substantial variability in differentially expressed genes across studies, with only modest overlap at the single-gene level but greater convergence at the pathway level (Baalbaki et al., 2025). Similarly, multi-cohort analyses have shown that long COVID is characterized by biologically heterogeneous molecular subtypes, with divergent transcriptomic profiles depending on severity and phenotype (Ai et al., 2025).

Despite these limitations, several immune-related genes (*CD4, IL6R, CD163, LAMP1, NFATC1, HLA-E*) and key metabolic–inflammatory features contributing to necroptosis and serotonergic synapse pathways showed consistent directionality across datasets, providing modest pathway-level concordance. This pattern aligns with prior evidence that biologically meaningful signals in heterogeneous post-infectious conditions are more robustly captured at the pathway or network level rather than at individual gene resolution (Baalbaki et al., 2025).

## 5 Limitations

The small sample size limits statistical power, increases the risk of overfitting in high-dimensional analyses, and may inflate effect size estimates. Accordingly, this pilot integrative analysis should be considered exploratory and hypothesis-generating. In addition, differences in matching and sample selection strategies between transcriptomic and metabolomic analyses, driven by biospecimen availability and quality constraints, resulted in differences in sex distribution across analytical platforms. Although age and sex were balanced between LC cases and controls within each analysis, these design differences may introduce heterogeneity between platforms and represent an additional study limitation. Serum metabolomic profiles may also be influenced by covariates such as diet, fasting status, circadian variation, medication use, and physical activity. Because previously biobanked samples were used, detailed metadata for several of these variables were not available. Although age, sex, BMI, and sampling time were balanced between groups, residual metabolic confounding cannot be excluded. Cross-cohort validation was further limited by substantial methodological and clinical heterogeneity across publicly available LC datasets, likely contributing to reduced statistical replication. Therefore, the identified immune–metabolic signatures should be interpreted cautiously as candidate biomarkers requiring validation in larger, better-matched independent cohorts.

## 6 Conclusion and implications

Despite these limitations, this study highlights coordinated immune and metabolic alterations in non-hospitalized healthcare workers with LC. The data suggest partially distinct but interacting immune programs, including neutrophil-associated inflammatory signatures and NK-linked regulatory pathways, alongside metabolic perturbations involving amino acid and energy metabolism. Overall, these findings support immune–metabolic dysregulation in LC as a systems-level phenomenon, while remaining exploratory. Larger, longitudinal, and standardized multi-cohort studies will be required to validate these signatures and clarify their clinical relevance.

## Data Availability

The datasets presented in this study can be found in online repositories. The names of the repository/repositories and accession number(s) can be found below: https://github.com/estefespin/CORSIP _omics, Github.
