# Supplementary material for: Pilot longitudinal integrated transcriptomic–metabolomic study reveals immune and metabolic signatures in non-hospitalized healthcare workers with long COVID
Source: Front Cell Infect Microbiol. 2026 Jun 4;16:1808564. doi: 10.3389/fcimb.2026.1808564 (PMC13275656; doi:10.3389/fcimb.2026.1808564)
Supplement: Supplementary file 1 [file Table1.docx]

**Supplementary Table 1. Predefined NanoString immune functional modules used to derive transcriptional scores, and Spearman correlations between these scores and selected immune markers (*S100A8* and *KIR2DL1*). Correlation coefficients (ρ), p values, and significance (S) are reported.**

| **Predefined NanoString Immune Functional Modules** | | | | |
| --- | --- | --- | --- | --- |
| **Modules** | **Transcriptional Score** | **Genes** | | |
| NK Regulatory Module | NK score | *IL21R, KIR2DL3, KIR3DL1, KIR3DL2, NCR1, XCL2* | | |
|  | NK function score | *CCR1, CD2, CD7, CXCL11, CXCR3, IFNG, IL12A, IL12B, IL12RB1, IL12RB2, IL18, IL18R1, IL18RAP, IRF1, ITGA1, KIR3DS1, KIR2DS1, KIR2DL1, KIR2DL3, KIR3DL1, KIR3DL2, KIR3DL3, KLRB1, KLRC1, KLRC2, KLRD1, KLRF1, KLRG1, KLRK1, LILRB1, NCR1* | | |
| Cytotoxicity Module | Cytotoxicity score | *GNLY, GZMA, GZMB, GZMH, GZMK, GZMM, HLA.A, HLA.B, HLA.C, PRF1* | | |
| Inflammatory Module | Neutrophil score | *CSF3R, FCGR3A, S100A12* | | |
|  | Monocyte/Macrophage score | *CD163, CD68, CD84* | | |
| **Spearman Correlation** | | **S** | **p value** | **ρ** |
| *S100A8* and Neutrophil_score | | 2678 | 0.0000 | 0.6826 |
| *S100A8* and Monocyte_score | | 10706 | 0.1073 | -0.2691 |
| *S100A8* and Cytotoxicity_score | | 8568 | 0.9269 | -0.0156 |
| *S100A8* and NK_function_score | | 9726 | 0.3648 | -0.1529 |
| *KIR2DL1* and NK_score | | 1568 | 0.0000 | 0.8141 |
| *KIR2DL1* and NK_function_score | | 3572 | 0.0002 | 0.5766 |
| *KIR2DL1* and Cytotoxicity_score | | 4824 | 0.0087 | 0.4282 |
| *KIR2DL1* and Neutrophil_score | | 11732 | 0.0174 | -0.3907 |
| *KIR2DL1* and *S100A8* | | 12450 | 0.0032 | -0.4758 |
