# Supplementary material for: Pilot longitudinal integrated transcriptomic–metabolomic study reveals immune and metabolic signatures in non-hospitalized healthcare workers with long COVID
Source: Front Cell Infect Microbiol. 2026 Jun 4;16:1808564. doi: 10.3389/fcimb.2026.1808564 (PMC13275656; doi:10.3389/fcimb.2026.1808564)
Supplement: Supplementary file 2 [file Table2.docx]

**Supplementary Table 2. Differentially abundant metabolites in LC cases versus controls at the post-infection timepoint, identified using a two-tailed t-test (p < 0.05) and limma with FDR correction, reported with Cohen’s d effect sizes.**

| **t.stat** | **P value** | **Metabolites** | **Log FC** | **Average Expression** | **P value** | **FDR< 0.05** | **Cohen’s d** |
| --- | --- | --- | --- | --- | --- | --- | --- |
| 4.6768 | 0.0009 | 1,2-dilinoleoylglycerol | 2.854 | 7.769 | <0.001 | 0.009 | 2.700 |
| 2.8419 | 0.0175 | 1,2-dihexadecanoyl-sn-glycero-3-phosphocholine | 1.027 | 14.302 | 0.012 | 0.044 | 1.641 |
| -2.365 | 0.0396 | 2,3-dihydroxybenzoate | -2.348 | 9.808 | 0.038 | 0.044 | -1.270 |
| 2.4853 | 0.0322 | butanoic acid, 4-[(1,2-dioxohexadecyl)amino]-, ethyl ester | 2.302 | 8.025 | 0.022 | 0.044 | 1.432 |
| 2.4688 | 0.0332 | deoxyuridine | 1.470 | 11.631 | 0.023 | 0.044 | 1.425 |
| -2.3966 | 0.0375 | glutamine | -1.561 | 9.509 | 0.027 | 0.044 | -1.384 |
| 2.3819 | 0.0385 | hypoxanthine | 0.625 | 12.787 | 0.032 | 0.044 | 1.375 |
| -3.3103 | 0.0079 | indole-3-acetate | -0.630 | 13.546 | 0.007 | 0.044 | -1.911 |
| -2.2444 | 0.0486 | indole-3-methyl acetate | -2.229 | 7.922 | 0.035 | 0.044 | -1.296 |
| -2.8317 | 0.0178 | lauroylcarnitine | -2.604 | 7.945 | 0.011 | 0.044 | -1.647 |
| -2.5778 | 0.0275 | l-carnitine | -0.510 | 11.582 | 0.026 | 0.044 | -1.488 |
| -2.3657 | 0.0396 | n-acetylasparagine | -1.561 | 9.971 | 0.028 | 0.044 | -1.366 |
| 2.8372 | 0.0176 | n-acetylcysteine | 0.438 | 14.384 | 0.020 | 0.044 | 1.638 |
| -2.9055 | 0.0157 | n-acetylglucosamine | -0.961 | 15.136 | 0.011 | 0.044 | -1.677 |
| 3.0193 | 0.0129 | oxoadipate | 2.162 | 8.732 | 0.008 | 0.044 | 1.743 |
| 2.4087 | 0.0368 | oxoglutarate | 0.415 | 10.840 | 0.038 | 0.044 | 1.391 |
| -3.2334 | 0.009 | thiamine monophosphate | -0.391 | 11.544 | 0.014 | 0.044 | -1.867 |
| 2.889 | 0.0161 | threonine | 0.260 | 17.942 | 0.039 | 0.044 | 1.668 |
| 2.6227 | 0.0255 | tricosanoate | 0.509 | 12.970 | 0.024 | 0.044 | 1.514 |
| 2.3453 | 0.041 | tryptophan | 1.148 | 15.536 | 0.030 | 0.044 | 1.354 |
| 2.9554 | 0.0144 | ureidopropionate | 0.301 | 13.849 | 0.028 | 0.044 | 1.706 |
| 2.6256 | 0.0254 | allothreonine | 0.279 | 15.307 | 0.044 | 0.048 | 1.516 |
| 2.5065 | 0.0311 | asparagine | 0.282 | 12.131 | 0.049 | 0.051 | 1.447 |
| -2.6441 | 0.0246 | quinolinate | -0.217 | 10.794 | 0.064 | 0.064 | -1.527 |
