# Supplementary material for: Pilot longitudinal integrated transcriptomic–metabolomic study reveals immune and metabolic signatures in non-hospitalized healthcare workers with long COVID
Source: Front Cell Infect Microbiol. 2026 Jun 4;16:1808564. doi: 10.3389/fcimb.2026.1808564 (PMC13275656; doi:10.3389/fcimb.2026.1808564)
Supplement: Supplementary file 3 [file Table3.docx]

**Supplementary Table 3. Differentially expressed genes in LC cases versus controls, identified using limma with FDR correction and reported with Cohen’s d effect sizes.**

| **Gene** | **Full Name** | **Log Fold Change** | **P value** | **FDR < 0.10** | **Cohen’s d** |
| --- | --- | --- | --- | --- | --- |
| *ABL1* | c-abl oncogene 1, non-receptor tyrosine kinase | -0.338 | <0.001 | 0.017 | -1.758 |
| *ADGRE5* | CD97 molecule | -0.432 | <0.001 | 0.029 | -1.505 |
| *ANXA1* | annexin A1 | 0.586 | <0.001 | 0.017 | 1.603 |
| *APP* | amyloid beta (A4) precursor protein | -0.498 | <0.001 | 0.029 | -1.448 |
| *ATF2* | activating transcription factor 2 | 0.226 | <0.001 | 0.017 | 1.951 |
| *ATG5* | autophagy related 5 | 0.361 | <0.001 | 0.029 | 1.456 |
| *BATF* | basic leucine zipper transcription factor, ATF-like | 0.303 | 0.004 | 0.072 | 1.212 |
| *BID* | BH3 interacting domain death agonist | 0.292 | 0.008 | 0.099 | 1.110 |
| *BMI1* | BMI1 polycomb ring finger oncogene | 0.239 | 0.008 | 0.097 | 1.152 |
| *BTLA* | B and T lymphocyte associated | 0.400 | 0.001 | 0.034 | 1.401 |
| *CD163* | CD163 molecule | -0.702 | <0.001 | 0.029 | -1.413 |
| *CD164* | CD164 molecule, sialomucin | 0.276 | 0.004 | 0.069 | 1.238 |
| *CD2* | CD2 molecule | 0.247 | 0.007 | 0.096 | 1.152 |
| *CD37* | CD37 molecule | -0.291 | 0.003 | 0.058 | -1.277 |
| *CD3D* | CD3d molecule, delta (CD3-TCR complex) | 0.521 | <0.001 | 0.023 | 1.528 |
| *CD4* | CD4 molecule | -0.362 | 0.003 | 0.056 | -1.271 |
| *CD47* | CD47 molecule | 0.261 | <0.001 | 0.029 | 1.557 |
| *CD48* | CD48 molecule | 0.435 | 0.002 | 0.044 | 1.306 |
| *CD5* | CD5 molecule | -0.271 | 0.005 | 0.078 | -1.208 |
| *CD58* | CD58 molecule | 0.417 | <0.001 | 0.014 | 1.837 |
| *CD7* | CD7 molecule | -0.395 | <0.001 | 0.017 | -1.670 |
| *CD74* | CD74 molecule, major histocompatibility complex, class II invariant chain | -0.320 | 0.004 | 0.066 | -1.234 |
| *CSF1* | colony stimulating factor 1 (macrophage) | -0.445 | 0.003 | 0.059 | -1.226 |
| *GPI* | glucose-6-phosphate isomerase | -0.299 | <0.001 | 0.029 | -1.539 |
| *HLA-A* | major histocompatibility complex, class I, A | -0.298 | 0.005 | 0.081 | -1.185 |
| *HLA-B* | major histocompatibility complex, class I, B | -0.390 | <0.001 | 0.017 | -1.671 |
| *HLA-E* | major histocompatibility complex, class I, E | -0.266 | 0.002 | 0.044 | -1.383 |
| *HMGB1* | high mobility group box 1 | 0.350 | 0.002 | 0.046 | 1.312 |
| *IFNGR1* | interferon gamma receptor 1 | 0.299 | 0.006 | 0.090 | 1.152 |
| *IGF2R* | insulin-like growth factor 2 receptor | -0.549 | 0.002 | 0.046 | -1.277 |
| *IL17RA* | interleukin 17 receptor A | -0.360 | 0.007 | 0.095 | -1.120 |
| *IL2RB* | interleukin 2 receptor, beta | -0.422 | 0.008 | 0.099 | -1.091 |
| *IL6R* | interleukin 6 receptor | -0.351 | 0.008 | 0.099 | -1.095 |
| *INPP5D* | inositol polyphosphate-5-phosphatase, 145kDa | -0.235 | 0.002 | 0.040 | -1.459 |
| *ITGA2B* | integrin, alpha 2b (platelet glycoprotein IIb of IIb/IIIa complex, antigen CD41) | -1.223 | <0.001 | 0.029 | -1.414 |
| *ITGA5* | integrin, alpha 5 (fibronectin receptor, alpha polypeptide) | -0.380 | <0.001 | 0.030 | -1.439 |
| *ITGAE* | integrin, alpha E (antigen CD103, human mucosal lymphocyte antigen 1; alpha polypeptide) | 0.411 | <0.001 | 0.017 | 1.762 |
| *ITGAM* | integrin, alpha M (complement component 3 receptor 3 subunit) | -0.425 | 0.001 | 0.040 | -1.346 |
| *JAK1* | Janus kinase 1 | -0.286 | <0.001 | 0.029 | -1.529 |
| *KIR2DL1* | killer cell immunoglobulin-like receptor, two domains, long cytoplasmic tail, 1 | -0.652 | 0.003 | 0.058 | -1.216 |
| *LAMP1* | lysosomal-associated membrane protein 1 | -0.379 | <0.001 | 0.014 | -1.860 |
| *LY96* | lymphocyte antigen 96 | 0.633 | 0.008 | 0.099 | 1.075 |
| *MAP2K1* | mitogen-activated protein kinase kinase 1 | -0.235 | 0.004 | 0.067 | -1.285 |
| *MAP3K1* | mitogen-activated protein kinase kinase kinase 1, E3 ubiquitin protein ligase | -0.232 | 0.003 | 0.058 | -1.340 |
| *MAP3K7* | mitogen-activated protein kinase kinase kinase 7 | 0.124 | 0.007 | 0.095 | 1.571 |
| *MAVS* | mitochondrial antiviral signaling protein | -0.212 | 0.002 | 0.041 | -1.483 |
| *NFATC1* | nuclear factor of activated T-cells, cytoplasmic, calcineurin-dependent 1 | -0.359 | <0.001 | 0.002 | -2.368 |
| *NOTCH1* | notch 1 | -0.448 | 0.006 | 0.085 | -1.138 |
| *PIK3CD* | phosphatidylinositol-4,5-bisphosphate 3-kinase, catalytic subunit delta | -0.349 | <0.001 | 0.029 | -1.473 |
| *REL* | v-rel reticuloendotheliosis viral oncogene homolog (avian) | 0.178 | 0.005 | 0.078 | 1.335 |
| *RELA* | v-rel reticuloendotheliosis viral oncogene homolog A (avian) | -0.269 | <0.001 | 0.023 | -1.668 |
| *RPS6* | ribosomal protein S6 | 0.709 | <0.001 | 0.022 | 1.540 |
| *S100A8* | S100 calcium binding protein A8 | 1.149 | 0.001 | 0.040 | 1.322 |
| *SELPLG* | selectin P ligand | -0.288 | 0.002 | 0.041 | -1.382 |
| *STAT3* | signal transducer and activator of transcription 3 (acute-phase response factor) | -0.290 | 0.006 | 0.085 | -1.173 |
| *TANK* | TRAF family member-associated NFKB activator | 0.362 | 0.001 | 0.040 | 1.368 |
| *TAP2* | transporter 2, ATP-binding cassette, sub-family B (MDR/TAP) | -0.452 | 0.004 | 0.068 | -1.193 |
| *TAPBP* | TAP binding protein (tapasin) | -0.370 | 0.002 | 0.040 | -1.358 |
| *TCF7* | transcription factor 7 (T-cell specific, HMG-box) | -0.284 | 0.007 | 0.096 | -1.132 |
| *TGFB1* | transforming growth factor, beta 1 | -0.431 | <0.001 | 0.031 | -1.410 |
| *THBS1* | thrombospondin 1 | -0.836 | 0.003 | 0.057 | -1.224 |
| *TNFRSF1B* | tumor necrosis factor receptor superfamily, member 1B | -0.320 | 0.006 | 0.085 | -1.158 |
| *XCL2* | chemokine (C motif) ligand 2 | 0.499 | 0.007 | 0.096 | 1.094 |
