# Supplementary material for: Pilot longitudinal integrated transcriptomic–metabolomic study reveals immune and metabolic signatures in non-hospitalized healthcare workers with long COVID
Source: Front Cell Infect Microbiol. 2026 Jun 4;16:1808564. doi: 10.3389/fcimb.2026.1808564 (PMC13275656; doi:10.3389/fcimb.2026.1808564)
Supplement: Supplementary file 4 [file Table4.docx]

**Supplementary Table 4. Longitudinal metabolite changes from pre- to post-infection in LC cases and controls assessed by linear mixed-effects models (p < 0.05)**

| **Metabolite** | **p_value_interaction** | **p_adj_FDR_interaction** |
| --- | --- | --- |
| oxoglutarate | 0.001 | 0.023 |
| oxoadipate | 0.016 | 0.195 |
| threonine | 0.033 | 0.264 |
