# Supplementary material for: Pilot longitudinal integrated transcriptomic–metabolomic study reveals immune and metabolic signatures in non-hospitalized healthcare workers with long COVID
Source: Front Cell Infect Microbiol. 2026 Jun 4;16:1808564. doi: 10.3389/fcimb.2026.1808564 (PMC13275656; doi:10.3389/fcimb.2026.1808564)
Supplement: Supplementary file 5 [file Table5.docx]

**Supplementary table 5. KEGG pathway enrichment analysis of integrated transcriptomic and metabolomic data (FDR < 0.05)**

| **Pathway** | **Total** | **Expected** | **Hits** | **P.Value** | **FDR** |
| --- | --- | --- | --- | --- | --- |
| Alanine, aspartate and glutamate metabolism | 36 | 0.298 | 8 | 3.52e-10 | 1.12e-07 |
| Arginine biosynthesis | 21 | 0.174 | 6 | 1.24e-08 | 1.97e-06 |
| Th17 cell differentiation | 107 | 0.885 | 9 | 1.93e-07 | 2.04e-05 |
| Hepatitis B | 163 | 1.35 | 10 | 7.43e-07 | 5.91e-05 |
| HTLV-I infection | 219 | 1.81 | 11 | 1.45e-06 | 9.19e-05 |
| Carbon metabolism | 116 | 0.96 | 8 | 4.41e-06 | 0.000212 |
| Epstein-Barr virus infection | 201 | 1.66 | 10 | 4.99e-06 | 0.000212 |
| D-Glutamine and D-glutamate metabolism | 5 | 0.0414 | 3 | 5.34e-06 | 0.000212 |
| Osteoclast differentiation | 128 | 1.06 | 8 | 9.17e-06 | 0.000304 |
| Th1 and Th2 cell differentiation | 92 | 0.761 | 7 | 9.56e-06 | 0.000304 |
| RIG-I-like receptor signaling pathway | 70 | 0.579 | 6 | 2.19e-05 | 0.000588 |
| Tryptophan metabolism | 42 | 0.347 | 5 | 2.22e-05 | 0.000588 |
| Antigen processing and presentation | 77 | 0.637 | 6 | 3.78e-05 | 0.000925 |
| Necroptosis | 162 | 1.34 | 8 | 5.08e-05 | 0.00115 |
| Kaposi's sarcoma-associated herpesvirus infection | 186 | 1.54 | 8 | 0.000134 | 0.00285 |
| HIF-1 signaling pathway | 100 | 0.827 | 6 | 0.000164 | 0.00326 |
| Inflammatory bowel disease (IBD) | 65 | 0.538 | 5 | 0.000186 | 0.00349 |
| TNF signaling pathway | 110 | 0.91 | 6 | 0.000276 | 0.00488 |
| Toxoplasmosis | 113 | 0.935 | 6 | 0.00032 | 0.0052 |
| Nitrogen metabolism | 17 | 0.141 | 3 | 0.000338 | 0.0052 |
| Leishmaniasis | 74 | 0.612 | 5 | 0.000343 | 0.0052 |
| Pancreatic cancer | 75 | 0.62 | 5 | 0.000365 | 0.00528 |
| EGFR tyrosine kinase inhibitor resistance | 79 | 0.653 | 5 | 0.000465 | 0.00643 |
| MAPK signaling pathway | 295 | 2.44 | 9 | 0.000655 | 0.00869 |
| Natural killer cell mediated cytotoxicity | 131 | 1.08 | 6 | 0.000705 | 0.00896 |
| Proximal tubule bicarbonate reclamation | 23 | 0.19 | 3 | 0.00085 | 0.0104 |
| Measles | 138 | 1.14 | 6 | 0.000926 | 0.0109 |
| Hematopoietic cell lineage | 97 | 0.802 | 5 | 0.00119 | 0.0133 |
| Pyrimidine metabolism | 57 | 0.472 | 4 | 0.00121 | 0.0133 |
| Viral carcinogenesis | 201 | 1.66 | 7 | 0.00127 | 0.0135 |
| T cell receptor signaling pathway | 101 | 0.835 | 5 | 0.00142 | 0.0146 |
| Metabolic pathways | 1430 | 11.8 | 22 | 0.00179 | 0.0177 |
| Citrate cycle (TCA cycle) | 30 | 0.248 | 3 | 0.00187 | 0.018 |
| Cellular senescence | 160 | 1.32 | 6 | 0.00198 | 0.0186 |
| PI3K-Akt signaling pathway | 354 | 2.93 | 9 | 0.00235 | 0.0214 |
| Serotonergic synapse | 115 | 0.951 | 5 | 0.00252 | 0.0223 |
| Cytokine-cytokine receptor interaction | 294 | 2.43 | 8 | 0.00275 | 0.0236 |
| Biosynthesis of amino acids | 75 | 0.62 | 4 | 0.00334 | 0.0276 |
| NOD-like receptor signaling pathway | 178 | 1.47 | 6 | 0.00338 | 0.0276 |
| Chronic myeloid leukemia | 76 | 0.629 | 4 | 0.0035 | 0.0278 |
