# Supplementary material for: Pilot longitudinal integrated transcriptomic–metabolomic study reveals immune and metabolic signatures in non-hospitalized healthcare workers with long COVID
Source: Front Cell Infect Microbiol. 2026 Jun 4;16:1808564. doi: 10.3389/fcimb.2026.1808564 (PMC13275656; doi:10.3389/fcimb.2026.1808564)
Supplement: Supplementary file 6 [file Table6.docx]

**Supplementary Table 6. Concordance in the direction of differential gene expression between the discovery cohort and validation cohorts A and B. Color intensity reflects agreement across cohorts: full concordance between discovery and both validation cohorts, partial concordance between discovery and one validation cohort, and no concordance between discovery and either validation cohort.**

| **Gene** | **Validation Cohort A** | | | | **Validation Cohort B** | | | |
| --- | --- | --- | --- | --- | --- | --- | --- | --- |
|  | **log Fold Change** | **p val** | **FDR** | **Direction Consistency** | **log Fold Change** | **p val** | **FDR** | **Direction Consistency** |
| *CD4* | -0.18 | 0.186 | 1 | TRUE | -0.267 | 0.023 | 0.182 | TRUE |
| *IL6R* | -0.144 | 0.344 | 1 | TRUE | -0.251 | 0.041 | 0.248 | TRUE |
| *CD163* | -0.29 | 0.319 | 1 | TRUE | -0.352 | 0.156 | 0.532 | TRUE |
| *LAMP1* | -0.046 | 0.545 | 1 | TRUE | -0.092 | 0.279 | 0.705 | TRUE |
| *NFATC1* | -0.028 | 0.746 | 1 | TRUE | -0.014 | 0.867 | 0.987 | TRUE |
| *HLA-E* | -0.011 | 0.913 | 1 | TRUE | -0.011 | 0.9 | 0.987 | TRUE |
| *RELA* | 0.031 | 0.863 | 1 | FALSE | -0.07 | 0.449 | 0.828 | TRUE |
| *PIK3CD* | 0.098 | 0.478 | 1 | FALSE | -0.063 | 0.491 | 0.842 | TRUE |
| *CD48* | -0.1 | 0.336 | 1 | FALSE | 0.015 | 0.884 | 0.987 | TRUE |
| *CD5* | 0.029 | 0.902 | 1 | FALSE | -0.018 | 0.911 | 0.987 | TRUE |
| *HMGB1* | -0.001 | 0.99 | 1 | FALSE | 0.006 | 0.962 | 0.987 | TRUE |
| *JAK1* | -0.024 | 0.734 | 1 | TRUE | 0.192 | 0.020 | 0.182 | FALSE |
| *BTLA* | 0.306 | 0.19 | 1 | TRUE | -0.070 | 0.643 | 0.965 | FALSE |
| *STAT3* | -0.041 | 0.751 | 1 | TRUE | 0.066 | 0.438 | 0.828 | FALSE |
| *TNFRSF1B* | -0.14 | 0.502 | 1 | TRUE | 0.093 | 0.535 | 0.856 | FALSE |
| *TCF7* | -0.055 | 0.704 | 1 | TRUE | 0.003 | 0.987 | 0.987 | FALSE |
| *ABL1* | -0.025 | 0.816 | 1 | TRUE |  |  |  |  |
| *CD58* | 0.024 | 0.848 | 1 | TRUE |  |  |  |  |
| *ATF2* | 0.038 | 0.763 | 1 | TRUE |  |  |  |  |
| *CD7* | -0.081 | 0.601 | 1 | TRUE |  |  |  |  |
| *HLA-B* | -0.085 | 0.529 | 1 | TRUE |  |  |  |  |
| *GPI* | -0.116 | 0.158 | 1 | TRUE |  |  |  |  |
| *APP* | -0.108 | 0.332 | 1 | TRUE |  |  |  |  |
| *CD47* | 0.119 | 0.186 | 1 | TRUE |  |  |  |  |
| *ITGAM* | -0.139 | 0.28 | 1 | TRUE |  |  |  |  |
| *SELPLG* | -0.108 | 0.616 | 1 | TRUE |  |  |  |  |
| *MAVS* | -0.013 | 0.937 | 1 | TRUE |  |  |  |  |
| *IGF2R* | -0.063 | 0.653 | 1 | TRUE |  |  |  |  |
| *THBS1* | -0.07 | 0.872 | 1 | TRUE |  |  |  |  |
| *KIR2DL1* | -0.664 | 0.093 | 1 | TRUE |  |  |  |  |
| *CD74* | -0.034 | 0.802 | 1 | TRUE |  |  |  |  |
| *MAP2K1* | -0.071 | 0.482 | 1 | TRUE |  |  |  |  |
| *CD164* | 0.018 | 0.863 | 1 | TRUE |  |  |  |  |
| *REL* | 0.478 | 0.073 | 1 | TRUE |  |  |  |  |
| *HLA-A* | -0.087 | 0.414 | 1 | TRUE |  |  |  |  |
| *IFNGR1* | 0.021 | 0.906 | 1 | TRUE |  |  |  |  |
| *IL17RA* | -0.112 | 0.402 | 1 | TRUE |  |  |  |  |
| *XCL2* | 0.12 | 0.59 | 1 | TRUE |  |  |  |  |
| *BMI1* | 0.039 | 0.707 | 1 | TRUE |  |  |  |  |
| *ITGAE* | -0.072 | 0.646 | 1 | FALSE | -0.161 | 0.294 | 0.705 | FALSE |
| *CD3D* | -0.238 | 0.074 | 1 | FALSE | -0.211 | 0.123 | 0.532 | FALSE |
| *TGFB1* | 0.089 | 0.558 | 1 | FALSE | 0.121 | 0.177 | 0.532 | FALSE |
| *TAPBP* | 0.078 | 0.416 | 1 | FALSE | 0.034 | 0.790 | 0.987 | FALSE |
| *INPP5D* | 0.063 | 0.567 | 1 | FALSE | 0.127 | 0.148 | 0.532 | FALSE |
| *CSF1* | 0.258 | 0.233 | 1 | FALSE | 0.035 | 0.858 | 0.987 | FALSE |
| *BATF* | -0.064 | 0.688 | 1 | FALSE | -0.393 | 0.009 | 0.182 | FALSE |
| *BID* | -0.145 | 0.458 | 1 | FALSE | -0.137 | 0.333 | 0.727 | FALSE |
| *ANXA1* | -0.041 | 0.814 | 1 | FALSE |  |  |  |  |
| *RPS6* | -0.162 | 0.213 | 1 | FALSE |  |  |  |  |
| *ADGRE5* | 0.018 | 0.883 | 1 | FALSE |  |  |  |  |
| *ITGA2B* | 0.441 | 0.068 | 1 | FALSE |  |  |  |  |
| *ATG5* | -0.073 | 0.49 | 1 | FALSE |  |  |  |  |
| *ITGA5* | 0.034 | 0.839 | 1 | FALSE |  |  |  |  |
| *S100A8* | -0.017 | 0.946 | 1 | FALSE |  |  |  |  |
| *TANK* | -0.023 | 0.882 | 1 | FALSE |  |  |  |  |
| *MAP3K1* | 0.16 | 0.24 | 1 | FALSE |  |  |  |  |
| *CD37* | 0.031 | 0.787 | 1 | FALSE |  |  |  |  |
| *TAP2* | 0.092 | 0.243 | 1 | FALSE |  |  |  |  |
| *NOTCH1* | 0.103 | 0.47 | 1 | FALSE |  |  |  |  |
| *MAP3K7* | -0.035 | 0.579 | 1 | FALSE |  |  |  |  |
| *CD2* | -0.012 | 0.924 | 1 | FALSE |  |  |  |  |
| *IL2RB* | 0.008 | 0.965 | 1 | FALSE |  |  |  |  |
| *LY96* | -0.033 | 0.83 | 1 | FALSE |  |  |  |  |
