# Supplementary material for: Pilot longitudinal integrated transcriptomic–metabolomic study reveals immune and metabolic signatures in non-hospitalized healthcare workers with long COVID
Source: Front Cell Infect Microbiol. 2026 Jun 4;16:1808564. doi: 10.3389/fcimb.2026.1808564 (PMC13275656; doi:10.3389/fcimb.2026.1808564)
Supplement: Supplementary file 7 [file Table7.docx]

**Supplementary Table 7. Concordance in the direction of differential metabolite abundance between the discovery cohort and the validation cohort.**

| **Metabolite** | **Validation Cohort** | | | |
| --- | --- | --- | --- | --- |
|  | **Log Fold Change** | **p val** | **FDR** | **Direction Consistency** |
| asparagine | 0.267 | 0.017 | 0.117 | TRUE |
| tryptophan | 0.168 | 0.050 | 0.173 | TRUE |
| threonine | 0.090 | 0.340 | 0.701 | TRUE |
| l_carnitine | -0.062 | 0.515 | 0.701 | TRUE |
| indole_3_acetate | -0.073 | 0.577 | 0.701 | TRUE |
| oxoglutarate | 0.016 | 0.864 | 0.864 | TRUE |
| glutamine | 0.045 | 0.601 | 0.701 | FALSE |
