# Supplementary material for: Pilot longitudinal integrated transcriptomic–metabolomic study reveals immune and metabolic signatures in non-hospitalized healthcare workers with long COVID
Source: Front Cell Infect Microbiol. 2026 Jun 4;16:1808564. doi: 10.3389/fcimb.2026.1808564 (PMC13275656; doi:10.3389/fcimb.2026.1808564)
Supplement: Supplementary file 8 [file Table8.docx]

**Supplementary Table 8. Necroptosis and serotonergic synapse pathways enriched following metabolomics and transcriptomics integration in NetworkAnalyst. Metabolomic and transcriptomic hits contributing to these pathways with consistent direction of fold change across validation cohorts are indicated. Metabolomic hits were mapped to their corresponding enzyme-coding genes in MetaCyc.**

| **KEGG Pathways** | **Total** | **Expected** | **Hits** | **Metabolomics Hits** | **Transcriptomics Hits** | **P.Value** | **FDR** |
| --- | --- | --- | --- | --- | --- | --- | --- |
| Necroptosis | 162 | 1.76 | 9 | *GLUL* (glutamine***** metabolism; MetaCyc-annotated)  *GLUD1* (oxoglutarate***** metabolism; MetaCyc-annotated)  *GLUD2* (oxoglutarate***** metabolism; MetaCyc-annotated) | *STAT3****^A^****, HMGB1****^B^****, BID, JAK1****^A^****, CASP1, IFNGR1****^A^*** | <0.001 | <0.001 |
| Serotonergic synapse | 115 | 1.25 | 5 | *TPH1* (tryptophan***** metabolism; MetaCyc-annotated)  *DDC* (tryptophan***** metabolism; MetaCyc-annotated) | *APP****^A^****, CASP3, MAP2K1* | 0.008 | 0.034 |

* Consistent direction of fold change in the metabolomics validation cohort.

**^A^** Consistent direction of fold change in the transcriptomics validation cohort A.

**^B^** Consistent direction of fold change in the transcriptomics validation cohort B.
